# Quasi-bound states in the continuum driven photoresponse in multiple quantum wells for machine vision

**DOI:** 10.1038/s41377-026-02404-4

**Published:** 2026-07-03

**Authors:** Wenjuan Zhou, Jun Deng, Pengying Chang, Hongrui Dou, Boyu Yang, Yan Chen, Peipei Chen, Chen Xu, Jinchao Tong, Jianlu Wang, Yiyang Xie, Junhao Chu

**Affiliations:** 1 Key Laboratory of Optoelectronics Technology, Beijing University of Technology, Ministry of Education, 100124 Beijing, China; 2 Institute of Optoelectronics, College of Future Information Technology, College of Integrated Circuits & Micro-Nano Electronics, Fudan University, Shanghai, 200433 China; 3 Nanofabrication Laboratory, CAS Key Laboratory for Nanophotonic Materials and Devices, National Center for Nanoscience and Technology, 100190 Beijing, China

**Keywords:** Optical materials and structures, Metamaterials

## Abstract

Bound states in the continuum (BIC) leverage symmetry-protected resonant modes for exceptional light confinement, yet their leaky modes are almost underutilized. Meanwhile, multiple quantum well (MQW) structures face limited optical absorption due to strict transition selection rules. We demonstrate the regulation of the leaky mode of quasi-BIC (QBIC) by analyzing MQW-vertical field coupling, revealing that increasing asymmetric parameters enhances the transverse leakage of wave vector and optical field nonlinearly. This drives a nonlinear photoresponse as increasing asymmetry parameter, while linear scenario with incident angle and external bias voltage. We then develop an optoelectrical fusion neuromorphic processor, implementing QBIC-MQWs into an artificial neural network for machine vision applications.

**Figure:**
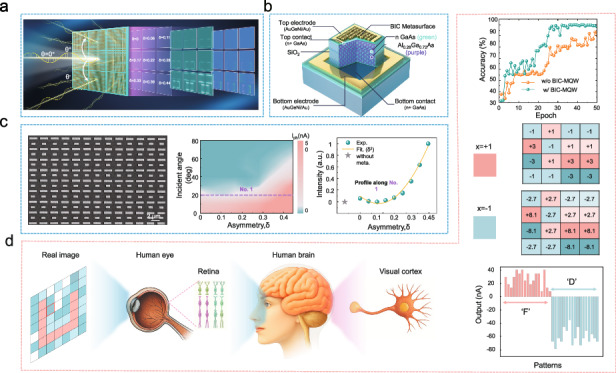
**Graphical abstract.** This work presents a proof-of-concept BIC-MQW device, in which the photocurrent characteristics are leveraged to realize image processing functionalities

**Graphical abstract.** This work presents a proof-of-concept BIC-MQW device, in which the photocurrent characteristics are leveraged to realize image processing functionalities

## Introduction

Metasurfaces enable subwavelength-pixelated light manipulation of amplitude, phase, polarization, and propagation, emerging as a transformative platform for tailoring light-matter interactions. This capability facilitates in-situ electromagnetic wave engineering in integrated optoelectronic systems, unlocking monolithic designs for advanced photonic processors^1–4^. Bound states in the continuum (BIC) that exploit nonradiating modes to achieve theoretically infinite quality factors (Q) and their quasi-BIC (QBIC) counterparts that emerge via symmetry-broken perturbations to enable high-Q leaky resonances, accompanied with unprecedented light confinement in subwavelength volumes, have received significant attention in nanoscale lasing^5–11^, biomolecular sensing^12–14^, optical imaging^15^, and other meta-devices^16–28^. These works primarily focus on the resonance modes arising from electromagnetic interference within periodic meta-atom arrays. The exploration and application of the symmetry-broken leaky modes generated by engineered radiation channels of QBIC remain relatively limited. According to Bloch theorem, electromagnetic modes can be represented by a wave vector ***k***_*|*| _= (***k***_*x*_, ***k***_*y*_) that is parallel to the *xy* plane. For symmetry-protected BICs, the radiation direction is purely normal to the *xy* plane, yielding ***k***_*|*|_≈0. Nevertheless, once the symmetry is broken, a structural perturbation that breaks the symmetry can transform a BIC into a QBIC, in which at least one component of the in-plane wave vector ***k***_*x*_ or ***k***_*y*_ is no longer being zero, giving rise to leaky modes generated by tailored QBIC meta-atoms, and generating energy transfer from leaky modes to free-space radiation fields through momentum conservation^29–33^. The wavevector components (***k***_*x*_, ***k***_*y*_), a fundamental degree of freedom for ***k***-space optical field manipulation, can establish a design paradigm in topology-optimized beam steering for ultracompact optics, nonlocal metasurface optics for aberration-free computational imaging, fano-enhanced near-field spectroscopy for single-molecule biosensing, and especially in subwavelength light-dielectric manipulation for design of novel optoelectrical devices.

Multiple quantum well (MQW) structures, comprising alternating layers of semiconductors such as GaAs/AlGaAs, InGaAs/InP, enable precise quantum confinement of carriers through bandgap engineering. By tailoring well widths and barrier heights, MQWs create discrete energy subbands that govern optoelectronic transitions. These structures have undergone pioneering deployment in light-emitting diodes (LEDs)^34,35^, semiconductor lasers^35–38^, optical modulators^39^, and infrared detectors^40–42^. Nevertheless, the optical absorption of MQW is intrinsically constrained by intersubband selection rules^43–47^. Under strict momentum matching between incident photons and carrier wavevectors, the photoresponse is proportional to normal electric field |***E***_*z*_ | ^2^ that is generated through the wavevector components (***k***_*x*_, ***k***_*y*_) in active region^47^. As the leaky modes existed in QBIC offers an approach to achieve the controllable modulation of (***k***_*x*_, ***k***_*y*_) via altering the asymmetry of meta-atom, integration of QBIC onto MQW structures may improve the optical absorption of traditional MQWs, providing a route towards advancing the integrated optoelectronic systems particularly for on-chip spectrum tailoring and polariton-assisted photoresponse.

**Fig. 1:**
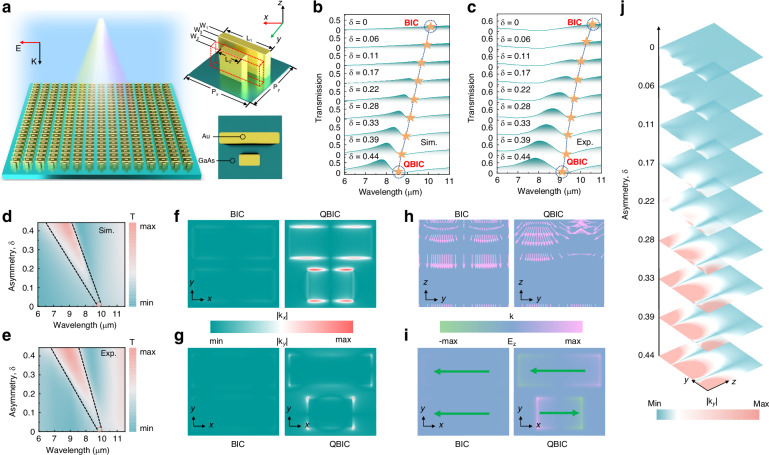
Characteristics of BIC. **a** Schematic diagram of the principle of BIC metasurfaces. The gold resonators (yellow), based on GaAs substrate (green), consist of two rectangular arms with lengths *L*_1_ and *L*_2_, widths *W*_1_ and *W*_2_, and a gap *W*_3_ between them. **b**–**e** Simulated and experimental transmission spectra of the metasurface. The dashed lines in Fig. 1d and e denote the transmission-peak profiles illustrated in Fig. 1b and c, respectively. **f**, **g** The wave vectors |***k***_*x*_| and |***k***_*y*_| in the *xy* plane, corresponding to the BIC and QBIC modes. **h** The wave vector ***k*** in the *yz* plane, corresponding to the BIC and QBIC modes. **i** The electric field strength and surface current in the *z*-direction, with green arrows representing the surface current direction on the *xy* plane. **j** The transverse leakage of the wave vector component |***k***_*y*_| corresponding to different asymmetry parameter δ in the simulation in the *yz* plane

This work presents the first demonstration that QBIC leaky modes can efficiently excite intersubband transitions in MQWs under normal incidence. The optical absorption within MQWs could be modulated by engineering the asymmetric parameters (δ from 0 to 0.44) of rectangular metal resonator metasurface, enabling a tunable nonlinear photoresponse directly harnessed for on-chip image sensing and contrast-enhancement preprocessing. While a linear photoresponse modulated by incident angle and external bias voltage allow a hardware implementation of a neural network for a high-level processing of image recognition and edge detection, respectively. In summary, our BIC-MQW platform offers three distinct advantages over existing technologies: (i) The first demonstration of QBIC-enabled normal-incidence operation in MQWs structure. (ii) A tunable nonlinear photoresponse directly applicable to machine vision tasks. (iii) Multifunctional operation (both linear and nonlinear) within a single device (see Supplementary Table S1).

## Results

### Design of BIC

A resonator composed of two rectangular metallic arms is designed as the fundamental unit of the metasurface to realize BIC or QBIC mode, as shown in Fig. 1a. The metallic component with 50 nm thick gold layer and 5 nm thick titanium adhesion layer is firstly deposited on GaAs substrate through magnetron sputtering and then is patterned using electron beam lithography (EBL) technique. Within the resonator, the lengths of the two arms are defined as *L*_1_ and *L*_2_, while their widths *W*_1_ and *W*_2_ are identical, and the distance of the two arms is represented by *W*_3_. The asymmetric parameter δ is defined as |*L*_1_-*L*_2_ | /*L*_1_, representing a key control variable that governs the structural deviation from equilibrium. If *L*_1_ is totally equivalent to *L*_2_ (i.e., δ = 0 case), the resonator works under BIC mode, otherwise under QBIC mode with δ ≠ 0 case. Herein, the *W*_1_ and *W*_2_ are set as 0.5 μm and the *W*_3_ is 0.7 μm, while the *L*_1_ is fixed with 1.8 μm and the *L*_2_ ranges from 1.8 to 1.0 μm, introducing a change in δ from 0 to 0.44. Specially, δ = 0 case corresponds to *L*_1_ = *L*_2_ = 1.8 μm, and δ = 0.44 case corresponds to *L*_1_ = 1.8 μm and *L*_2_ = 1.0 μm. The designed resonator is periodically distributed in *xy* plane with periods of *P*_*x*_ = *P*_*y*_ = 2.5 μm.

An electromagnetic simulation of our designed metasurface was performed using the Lumerical FDTD Solutions to analyze the mechanisms of symmetry-protected BIC mode and symmetry-broken QBIC mode, respectively (see “Materials and Methods” section). In the simulation, the metasurface is illuminated by a normally incident and linearly polarized plane wave characterized by the wave vector ***k*** = (0,0,-***k***_*z*_), and the electric field vector can be oriented along the *x*-axis (i.e., an *x*-polarized wave represented by ***E*** = (***E***_*x*_,0,0)). We focus on the transmission spectra around 8 μm in the LWIR range, which is consistent with the absorption wavelength of latter designed MQW (see Fig. 3e). Figure 1b, c demonstrates the simulated and the measured transmission spectra as a function of δ. In the case of δ = 0, the resonator exhibits a BIC mode, where electromagnetic energy is highly localized within the resonator core without leaking into free space, resulting in the disappearance of the resonance peak of transmission. As δ increases from 0 to 0.44, resonances of radiatively coupled QBIC begin to emerge, with an increase in amplitude and a blue shift in the peak position as well as an increase in full width at half maximum (FWHM) for the transmission spectra. Additionally, we separately calculated the Q-factors corresponding to Fig. 1b, c (see Fig. S1, also exhibiting similar trend between simulation and experiment.). These variations of transmission characteristics originating from the transitions between BIC and QBIC mode are also clearly presented in Fig. 1d, e, where transmission mappings with wavelength and δ from both simulation and experiment are provided respectively. It is obvious that these experimental results are in a good agreement with our simulations, but there are some tiny discrepancies in the resonance wavelength and bandwidth, which can be attributed to the manufacturing errors in metal and thus increased thermal loss, as well as distributed-angle illumination in experimental setup^48^.

The manipulation of the wave vector can be achieved by precisely controlling the leakage mode resulting from the breaking of structural symmetry. Figure 1f, g presents the strengths of wave vector component |***k***_*x*_| and |***k***_*y*_| in BIC mode (δ = 0) and QBIC mode (δ = 0.44 as an example), respectively. Under BIC mode, the strengths of |***k***_*x*_| and |***k***_*y*_| are almost zero, implying that the wave vectors are all directed along the *z*-axis and thus lateral energy flow into *xy* plane is suppressed due to symmetry constraints. That is to say, the electromagnetic waves that is primarily aligned along the *z*-axis forms a stationary wave field, leading to a disappear of in-plane radiation because of the lateral phase matching condition. In contrast, the QBIC mode exhibits the energy leakage into *xy* plane with the wave vectors extending beyond the *z*-axis direction, as the strong non-zero strengths of |***k***_*x*_| and |***k***_*y*_| are observed. Furthermore, Fig. 1h shows the corresponding direction of ***k*** in BIC mode and QBIC mode, respectively. The ***k*** mainly directs along the *z*-axis under BIC mode while deviates away from the *z*-axis under QBIC mode. The |***k***_*x*_| and |***k***_*y*_| in *xy* plane and the ***k*** direction in *yz* plane with δ (0 ~ 0.44) are seen in Supplementary Text part 1.1 and Figs. S2–S4 for details. These phenomenon on evolution of wave vectors confirm the suppression (or emergence) of energy leakage from *z*-axis into *xy*-plane in BIC (or QBIC) mode. Figure 1i gives the corresponding electric field strength ***E***_*z*_ (the evolution with varied δ see Fig. S5) and surface current in *z* direction in BIC mode and QBIC mode, respectively. There is negligible electric field and parallel current flowing for BIC mode, while a strong electric field and antiparallel current flowing are observed under QBIC mode. The emergence of such antiparallel current between two rectangular metallic arms indicates the formation of an electric dipole, which converts the localized energy into directed radiation electromagnetic waves under QBIC mode (for detailed analysis, see Supplementary Text part 1.2 and Fig. S6). In addition, the mapping of |***k***_*y*_| in *yz* plane with varied δ is shown in Fig. 1j. As δ increase, the antiparallel surface currents in resonator become stronger due to the enhanced transverse leakage of |***k***_*x*_| and |***k***_*y*_ | , as well as the electric field |***E***_*z*_ | .

**Fig. 2:**
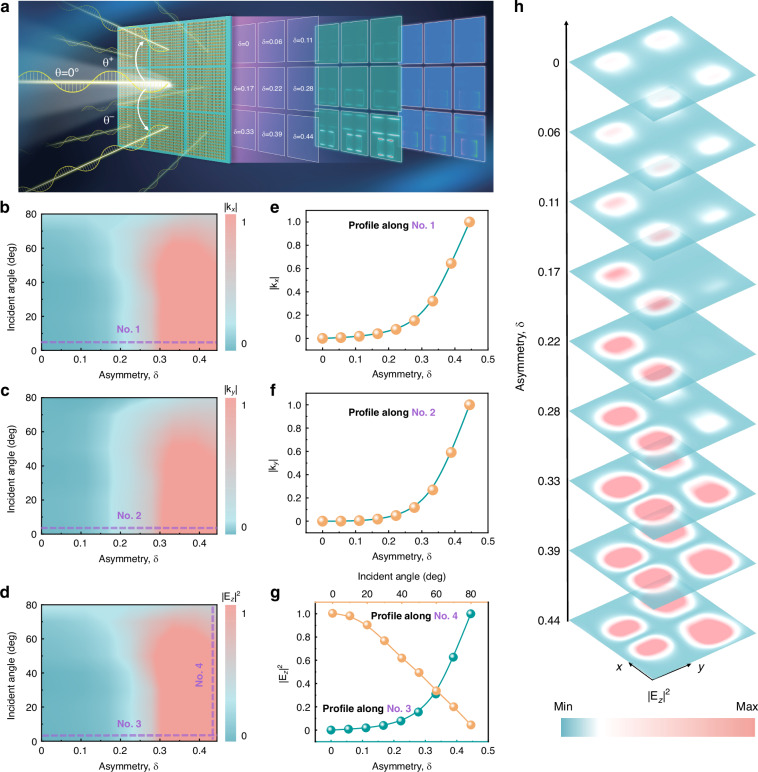
Simulation results at the origin position of MQW in BIC-MQW device. **a** Schematic illustration of simulation conditions. **b**, **c** The mapping relationship among the integral of lateral leaky components |***k***_*x*_| and |***k***_*y*_| of the wave vector, the asymmetry parameter δ, and the incident angles θ. **d** The mapping relationship among the integral of electric field |***E***_*z*_ | ^2^, the asymmetry parameter δ, and the incident angles θ. **e**–**g** The simulated results of integral of |***k***_*x*_ | , |***k***_*y*_| and |***E***_*z*_ | ^2^ on the *xy* plane respectively when the sample is illuminated vertically (i.e. θ = 0°), that is extracted along predetermined transects No.1-No.3 of the functional mapping (Fig. 2(b), (c) and (d)). The relationship between |***E***_*z*_ | ^2^ and θ is extracted along predetermined transects No.4 of the functional mapping (Fig. 2(d)). **h** The variations in electric field |***E***_*z*_ | ^2^ corresponding to different asymmetry parameters δ of θ = 0° on the *xy* plane at the origin position of the active region

To explore the potential couplings of BIC or QBIC with optoelectrical components, we performed a simulation of the BIC-MQW system, where the metasurface resonators are integrated on MQW structure. Figure 2a schematically illustrates the simulation framework to characterize the dependence of lateral leaky radiation on both δ of BIC structure and incident angle θ of incident waves entering MQWs structure. A comprehensive simulation of the evolution of the optical field (|***k***_*x*_ | , |***k***_*y*_| and |***E***_*z*_ | ^2^ as a function of δ and θ in *xy* plane) at various positions from the resonator to the active MQW structure is seen in Supplementary Text part 1.3 and Figs. S7–S8. The simulation results when the monitor was placed at the origin position of MQWs in BIC-MQW is discussed in the following. Figure 2b–d show the mappings of the integral of |***k***_*x*_ | , |***k***_*y*_*|*, and |***E***_*z*_ | ^2^ as a function of δ and θ, respectively. Meanwhile, the profiles along the cut lines labeled as No.1–4 in Fig. 2b–d are extracted as Fig. 2e–g. Figure 2h exhibits the evolution of |***E***_*z*_ | ^2^ in *xy* plane with δ at θ of 0°. It is evident that the magnitudes of |***k***_*x*_ | , |***k***_*y*_*|*, and |***E***_*z*_ | ^2^ all increase with δ in a nonlinear relationship, which is consistent with the previous findings about the mechanism between radiation losses and asymmetric parameters under QBIC model^49,50^. This nonlinear scaling originates from the fundamental symmetry-to-radiation transition during BIC-to-QBIC conversion. As δ increases, the broken symmetry transforms the ideal bound state into a leaky resonance whose radiation loss grows with δ, directly enhancing the in-plane wavevectors |***k***_*|*|_|. Concurrently, the enlarged |***k***_*|*|_| generates strong |***E***_*z*_| fields via polarization-singularity-enabled mode conversion, following the radiation fields |***E***_*z*_ | ²${\boldsymbol{\propto }}$δ². This radiation field enhancement activates normally forbidden intersubband transitions in MQWs. It is suggested that an increase in δ results in enlargement of the transverse leakage wave vector |***k***_*|*|_| due to enhancement of the electric field |***E***_*z*_| in a nonlinear relationship, during the transition from BIC to QBIC, thereby improving the coupling efficiency between the incident wave and the MQWs structure. Next, we performed quantitative calculations for bare-MQW and BIC-MQW to confirm the absorption enhancement inside the MQW region (see Supplementary Text part 1.4 and Fig. S9). We also evaluated the wavelength-dependent field enhancement by simulating |***E***_*z*_ | ^2^ within the active region for wavelengths from 6 μm to 10 μm, showing that integral of the |***E***_*z*_ | ^2^ exhibits pronounced nonlinear variation in the 7-8 μm range (see Fig. S10). Moreover, the magnitudes of |***k***_*x*_ | , |***k***_*y*_*|* and |***E***_*z*_ | ^2^ at δ = 0.44 all decrease with θ in a nearly linear relationship as seen in Fig. 2g. This is because angular increase introduces momentum mismatch, suppressing the conversion efficiency from ***k***_*|*|_ to ***E***_*z*_ nearly linearly because the efficiency of incident light is proportional to cosθ (the range of θ is from 0 to π/2)^51^. Consequently, these results manifest diverse functionalities in QBIC-MQW system due to the existence of both nonlinear and linear characteristics by changing δ and θ, providing an alternative approach to achieve the controllable modulation of photoresponse for optoelectronics application.

### Design and photocurrent characterization of BIC-MQWs

**Fig. 3:**
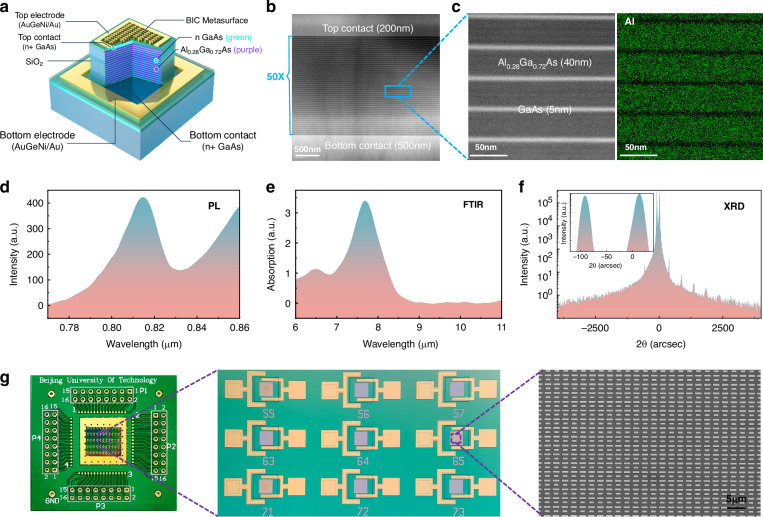
Design, characterization, and fabrication of BIC-MQWs device. **a** Single pixel integrated by BIC metasurface and MQWs. **b** Cross-sectional TEM image of entire MQWs. **c** Cross-sectional TEM image of localized MQWs and EDS elemental mapping of Al on MQWs structure. **d** The photoluminescence (PL) spectrum of BIC-MQWs device. **e** The absorption spectrum of the BIC-MQW device characterized via FTIR. **f** The XRD patterns of the sample. **g** The photograph of the photocurrent-programmable sample attached to the PCB with subsequent wire bonding, the optical micrograph of fabricated BIC-MQWs device, and SEM image of BIC metasurface

To verify the feasibility of the prediction above, a BIC-MQW system is experimentally demonstrated by integrating a BIC array with a size of 3 × 3 pixels onto the MQW devices, as depicted in Fig. 3 (for details, see “Materials and Methods”). The nine pixels correspond to nine values of δ as above mentioned, where each pixel composes 60 × 60 resonators as shown in Fig. 3a. The MQWs structure consists of 50-period alternating structure of GaAs/Al_0.28_Ga_0.72_As heterojunctions, of which cross-sectional TEM image is seen Fig. 3b (for theoretical analysis, see Fig. S11). To obtain a target absorption wavelength of 8 μm, the width of Al_0.28_Ga_0.72_As barrier is 40 nm and the width of the GaAs quantum well is approximately 5 nm, forming an MQW structure that exhibits an interband transition energy of 1.477 eV between the conduction and valence band (for detailed calculation, see Supplementary Text part 2)^52–54^. The electrochemical capacitance voltage (ECV) analysis results, illustrated in Fig. S12, indicate that the n-type doping concentration inside the quantum well can be precisely controlled during the preparation of MQWs (for details, see “Materials and Methods”). As magnified in Fig. 3c, cross-sectional TEM imaging reveals atomically high-quality interfaces between the 50-period GaAs/Al_0.28_Ga_0.72_As quantum wells, while ED–S elemental mapping further confirms precise compositional modulation with abrupt Al transitions due to exceptional epitaxial growth control. Next, we measured the photoluminescence (PL) spectrum^55^, as displayed in Fig. 3d. The PL peak wavelength is 0.816μm, corresponding to an energy difference of approximately 1.52 eV between the ground states of the electron and hole wells, which aligns with the design specifications of 1.477 eV. Figure 3e indicates the absorption spectrum of MQWs characterized via Fourier-transform infrared (FTIR) spectroscopy. The PL peak observed at ~0.816 μm arises from interband radiative recombination of electrons and holes between the conduction and valence band quantum well states, characterizing the electronic quality of the GaAs/AlGaAs material system. In contrast, the designed photoresponse in LWIR regime targets an intersubband transition (ISBT) within the conduction band of the same MQW, with an absorption peak near 7.7 μm, which is very close to the designed target of 8 μm. These two processes are orthogonal, involving different carrier populations and selection rules: interband transitions govern light emission/absorption at near-infrared telecommunication wavelengths, while ISBT enable mid- to long-infrared detection. The presented PL data thus serves to confirm the crystalline quality of the epitaxial layers, which is a prerequisite for high-performance ISBT-based devices. The X-ray diffraction (XRD) patterns of the sample are presented in Fig. 3f, which evidences that the Al_0.28_Ga_0.72_As layer maintains a kind lattice structure, with the GaAs and Al_0.28_Ga_0.72_As peaks separated by approximately 100 arcseconds. The numerous satellite peaks observed further support the high crystalline quality and good periodicity. Subsequently, the process flow for fabricating BIC-MQW sample see Fig. S13. The prepared BIC-MQW samples are evidenced by the photograph shown in Fig. 3g, and the microscope photo of the sample is shown in Figs. S14 and S15, which display the top-view morphology of the BIC metasurface, confirming the well-defined rectangular geometry of the fabricated metal structures. The experimental setup used to measure the photocurrent through the optical pathway is illustrated in Fig. S16. A beam of light generated by a QCL laser at a wavelength of 8 μm goes through a series of optical components, and a lens focuses the modulated light onto the BIC-MQW sample to ensure optimal-illumination and efficient light collection.

**Fig. 4:**
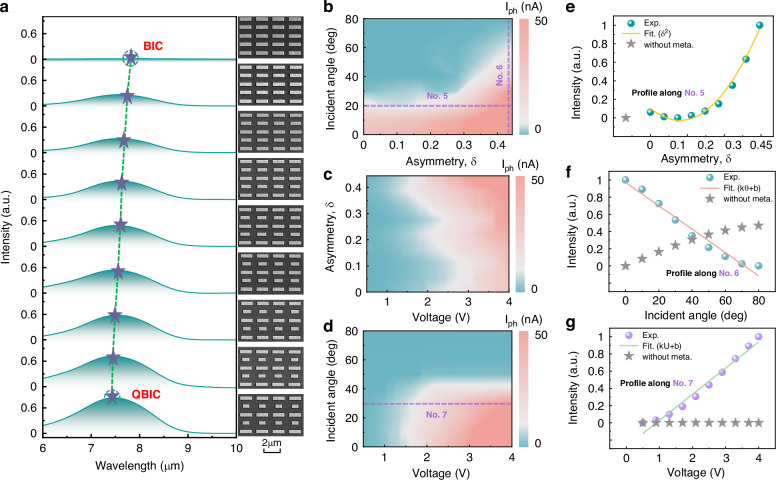
Experimental results of BIC-MQW device. **a** The spectral response of BIC-MQWs obtained through FTIR testing and SEM image of BIC metasurface corresponding to various asymmetric parameters. **b** The measured photocurrent as a function of δ and θ under same bias. **c** The measured photocurrent as a function of δ under different bias. **d** The measured photocurrent as a function of θ under different bias. **e** Characteristic profile was extracted along predetermined transects No.5 of the functional mapping (Fig. 4b). **f** Characteristic profile was extracted along predetermined transects No.6 of the functional mapping (Fig. 4b). **g** Characteristic profile was extracted along predetermined transects No.7 of the functional mapping (Fig. 4d) under normal incidence. The line charts in (**e**), (**f**), and (**g**) have been normalized along the *y*-axis. And the star symbols in Fig. (**e**), (**f**), and (**g**) denote the experimental data for the bare-MQW devices

The couplings of BIC-MQW system are further investigated by characterizing the photoresponse with and without BICs. The spectral response of the BIC-MQW devices with different δ were characterized via FTIR in Fig. 4a, and the corresponding SEM images of BIC metasurfaces are also shown. As δ increases, the enhancement of peak intensity accompanied with a blueshift of the response wavelength is experimentally observed in BIC-MQW device, which are consistent with that in solely BIC or QBIC metasurface (Fig. 1b, c). While the observed broadening of the spectral intensity range can be attributed to both the operating wavelength and the intrinsic material properties. Figure 4b depicts the measured photocurrent as a function of δ and θ, and the measurement system was displayed in Fig. S17. Characteristic profiles were extracted along the cut lines labeled as No.5 and No.6 in Fig. 4b and were plotted in Fig. 4e, f. Specifically, Fig. 4e demonstrates a nonlinear increase in photocurrent with rising δ, which is consistent with the results of the blackbody photocurrent measurement system (see Fig. S18 and Fig. S19), whereas Fig. 4f shows a linear decrease in photocurrent as θ increases (more detailed measurements, see Fig. S20). Note that nonlinear photoresponse with δ in BIC-MQW device well aligns with the optical response of leaky-mode QBICs (Fig. 2e–g), where enhancement of symmetry breaking can enhance the |*E*_*z*_ | ^2^ generated by radiative leakage and local field intensity. The linear photoresponse with θ in BIC-MQW device is also consistent with the |*E*_*z*_ | ^2^ variation in Fig. 2g. In addition, Fig. 4c, d presents the measured photocurrent of the BIC-MQW device under varied bias voltages, along with different δ and θ, respectively. Figure 4g illustrates the photocurrent as a function of applied voltage that is extracted from the cutline No.7 in Fig. 4d. It is obvious that photocurrent increases linearly with bias voltage. Herein, the threshold voltage greater than 0.5 V, so the linear characteristics are better after threshold. The observed linear dependence of photocurrent on θ arises from the small‑angle approximation over the measured angular range (see Table S2), while the linear photoresponse with bias voltages originates from the linear increase in carrier drift velocity with electric field in the low-field regime. Generally, by comparing the experimental data of BIC-MQW and bare-MQW devices in Fig. (e–g), we find that integrating BIC metasurfaces significantly enhances the optical absorption of MQW structures. Quantitatively speaking, the nonlinear photocurrent with δ from 0 to 0.44 can be featured by parabolic relationship with δ (i.e., photocurrent is proportional to δ^2^), while the linear characteristic is represented by kδ + b (k and b are constants). For nonlinear characteristics, the photocurrent in MQW device with BIC metasurface (δ = 0.44) is improved by 3.6 times as that without BIC metasurface, confirming the improved optical absorption by ${\boldsymbol{k}}$-space optical field manipulation. For linear characteristics, a dynamic ratio in linearly responded photocurrent is obtained to be 1.702 × 10^4^ as θ ranges between 0° and 80°, while it is 2.1867 × 10^2^ with external bias voltage from 0.5 to 4 V. Overall, the good agreements between experiment and simulation further verify the realization of asymmetrty-engineered BIC integrated on MQW structures, and successfully achieve the controllable modulation of nonlinear and linear photoresponse in BIC-MQW devices for optoelectronics based machine vison applications. All optoelectronic measurements were performed at 77 K using liquid nitrogen cooling, the standard operating temperature for long‑wave infrared MQWs that effectively suppresses the exponentially temperature‑dependent dark current^43^. Although this cryogenic operation is required for the present demonstration, recent advances in photon-engineering metasurfaces^40^ suggest that with appropriate design the operating temperature can be substantially increased, paving the way toward uncooled or moderately cooled in-sensor computing applications.

## Discussion

Machine vision is a technology that enables the recognition of visual information in a complicated environment, which consists of the visual sensing and preprocessing such as photoreception, contrast enhancement, denoising, and the further high-level post-processing, such as image recognition^56^. In the conventional machine vision system, the visual information is perceived by images sensors, converted to electrical signal, stored in memory units, and passed to processing units^57^. The data movement between separated sensing and processing units based on the von Neumann computing architectures suffers from high latency and high energy consumption^58^. The state-of-the-art near- or in-sensor neuromorphic computing architectures based on various optoelectronic devices have become a major research focus^59–61^. As demonstrated in Fig. 4, programable photoresponse of QBIC-MQWs can be adjusted by engineering the asymmetric parameters of BIC metasurface in a nonlinear way, and also can be optically or electrically tuned by incident angle of light shed on metasurface or bias voltage applied on MQW following a linear relationship. These versatile device characteristics offer the possibility of the hardware realization of multi-functionalities in image processing for a state-of-the-art visual system. Therefore, the 3 × 3 QBIC-MQW array is demonstrated to execute the image processing of contrast enhancement using the nonlinear photoresponse and perform the in-sensor multiply-and-accumulation (MAC) operation using the linear photoresponse (see Supplementary Text part 3 and Fig. S21–SFig. S23). Compared with conventional machine vision system, these in-sensor image preprocessing and computing can reduce the long-distance communication between sensory units and processing units, thereby enabling high-density integration by avoiding the complex processing circuits.

**Fig. 5:**
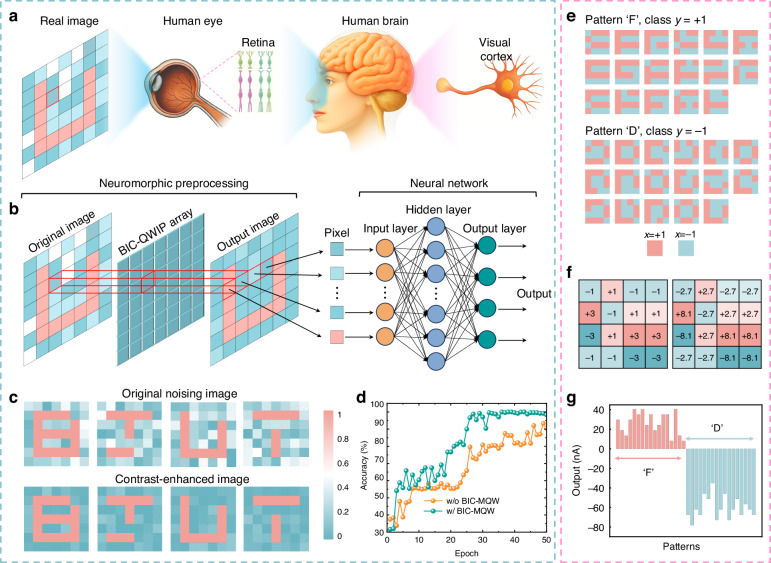
Simulations of image recognition in a neuromorphic visual system with BIC-MQW. **a** Illustration of the human visual system. **b** Schematics of an artificial neuromorphic visual system based on the BIC-MQW devices for image preprocessing and an artificial neural network for image recognition. **c** Comparison of images including letters ‘B’, ‘J’, ‘U’ and ‘T’ before and after BIC-MQW-based preprocessing: original noisy images (top row) and contrast-enhanced images (bottom row). **d** Recognition accuracy during training epochs with and without BIC-MQW-based preprocessing. **e** Two sets of patterns representing letters ‘F’ and ‘D’. **f** Calculated dimensionless weights and actual photoresponsivity weights in BIC-MQW. **g** Output currents during the presentations of ‘F’ and ‘D’ patterns

As contrast enhancement prior to recognition plays a vital role in image preprocessing for both human and machine vision, we performed simulations to emulate the hierarchical processing architecture in the human visual systems for more complex images, including the image preprocessing based on BIC-MQW arrays and the image recognition based on an artificial neural network. As shown in Fig. 5a, the human visual system consists of the retina and visual cortex, in which the retina performs the visual sensing and preprocessing such as photoreception, contrast enhancement, and denoising, while the visual cortex handles the further high-level post-processing such as image recognition. In the neuromorphic visual system as shown in Fig. 5b, BIC-MQW arrays are used to mimic the sensing and preprocessing functionality in the human retina, and the preprocessed images by the BIC-MQW array are then transmitted to an

artificial neural network to perform the image training and recognition tasks. A multilayer perceptron (MLP) neural network is constructed for recognizing the images. The MLP is a fully connected neural network, where each neuron node in one layer connects to every neuron node in the following layer. An image dataset that contains images of the letters ‘B’, ‘J’, ‘U’, and ‘T’ and their variants after adding noises was employed as both training and testing set. In our case, the MLP consists of an input layer (49), hidden layer (20) and output layer (4). The 49 input neurons correspond to 7 × 7 pixels of each images, while the 4 neurons of output layer correspond to 4 classes of letters ‘B’, ‘J’, ‘U’, and ‘T’. Detailed information of the dataset is provided in Supplementary Text part 4.1. One image example of each letter from the dataset were illustrated to compare the image contrast before and after preprocessing, as shown in Fig. 5c. According to the fitting curve of the experimental photocurrent-asymmetry relationship (see Fig. S24b) where input image pixels are mapped into asymmetry parameter and output image pixels are represented by photocurrent of BIC-MQW, the body pattern pixels of the letters were enhanced and the background pixels were weakened after the BIC-MQW-based preprocessing. The preprocessed image dataset was then input into the artificial neural network to implement the image training and recognition. Figure 5d illustrates the recognition results of artificial visual systems with and without BIC-MQW preprocessing, respectively. The accuracy of over 99% can be reached after 30 training epochs with BIC-MQW-based preprocessing, while a fluctuant accuracy of ~93% can be reached after 50 training epochs without BIC-MQW-based preprocessing. The recognition accuracies of different running cycles are presented in Supplementary Text part 4.2 and Fig. S25, implying the repeatability of the results. The recognition efficiency in convergence speed is listed in Supplementary Text part 4.3 and Table S3. Additionally, the wavelength-dependence recognition performance based on Fig. S10 is evaluated in Fig. S26. The robustness of BIC-MQW system against noise is also comprehensively explored and listed in Supplementary Text part 4.4 and Figs. S27-28. These simulation results prove obvious improvements in the recognition rate and efficiency with the treatment of contrast enhancement through BIC-MQW-based image preprocessing.

With the capability to conduct in-sensor MAC operation, the BIC-MQWs can be utilized to realize real-time image processing for pattern classification, another fundamental technique in computer vision and machine learning. Two sets of patterns denoting the letters ‘F’ and ‘D’ and their noisy versions are adopted as both training and test sets as shown in Fig. 5e, which consists of 50 patterns with some patterns appearing repeatedly several times (see Supplementary Text part 5.1 and Fig. S29). A single-layer perceptron, which has sixteen binary inputs corresponding to a 4 × 4 pixel array, one bias input *x*_0_ and one binary output *y*, is constructed to perform the pattern classification task (see Supplementary Text part 5.2). The input patterns are classified into two groups by conducting a weighted sum and a sign function operation: ${\boldsymbol{y}}={\boldsymbol{sgn}}{\boldsymbol{[}}{\sum }_{{\boldsymbol{i}}={\boldsymbol{0}}}^{{\boldsymbol{16}}}{{\boldsymbol{w}}}_{{\boldsymbol{i}}}{{\boldsymbol{x}}}_{{\boldsymbol{i}}}{\boldsymbol{]}}$, where weight *w*_*i*_ denotes a synaptic strength between the *i*-th input and the output (see Fig. S30). The inputs and outputs have the logical values + 1 or -1, whereas the bias is fixed with the logical values of +1. To execute the hardware implementation of pattern classification, the input image is mapped to the illumination conditions of the BIC-MQW, where the pixel value of +1 (or -1) is represented by applying (or removing) illumination stimuli with an optical power of ~ 2.58 μW to the corresponding BIC-MQW. The weight is mapped to the photoresponsivity of the BIC-MQW, which can be directly programed by the bias voltage that is applied to the BIC-MQW devices. The weights *w* that yield correct classification is obtained through the ex situ training process in software, and then the calculated weights were imported to the BIC-MQW. Figure 5f shows the calculated dimensionless weight matrix and the actual photoresponsivity weight matrix in unit of mA/W. Through the MAC operation of parallelly connected BIC-MQW, the BIC-MQW array generates an output current ${\boldsymbol{I}}={\boldsymbol{[}}{\sum }_{{\boldsymbol{i}}={\boldsymbol{0}}}^{{\boldsymbol{16}}}{{\boldsymbol{R}}}_{{\boldsymbol{i}}}{{\boldsymbol{P}}}_{{\boldsymbol{i}}}{\boldsymbol{]}}$ according to the Kirchhoff’s law, where *R*_*i*_ is the photoresponsivity of the BIC-MQW at the *i*-th pixel and *P*_*i*_ is the input optical power illuminated to the corresponding BIC-MQW. As shown in Fig. 5g, the output current is always positive when the input pattern belongs to the ‘F’ class, while it is always negative when the input pattern belongs to the ‘D’ class. Clearly, the input two patterns are well separated and the binary classification task reaches an accuracy of 100%. Furthermore, the edge detection via convolution neural network (CNN) is performed using the BIC-MQW where the kernel weight is also mapped to photoresponsivity but is modulated by the incident angel (see Supplementary Text part 6 and Fig. S32). The experiment demonstrates that the hardware implementation of real-time image processing such pattern classification and edge detection can be completed using the multifunctional BIC-MQW.

In summary, this work demonstrates a proof-of-concept BIC-MQW device, which proves that the leaky mode existed in QBICs improves optical absorption of MQWs through precisely modulating the asymmetry of rectangular meta-resonator. We designed and fabricated a Al_0.28_Ga_0.72_As/GaAs superlattice-based MQW with meta-resonator array integrated on top surface. The nonlinear photocurrent that is dependent on asymmetry parameter allows us to perform an image preprocessing of contrast enhancement to improve the efficiency and accuracy of subsequent recognition task, while the linear photocurrent that can be modulated by external bias voltage and incident angel of light is utilized to perform real-time image processing of pattern classification and edge detection. Except machine vision, this coexistence of enhanced intensity and spectral broadening (see Supplementary Text part 7 and Figs. S33–S34) also offer promising opportunities for device applications in broadband spectroscopic and multifunctional operation. Future work will focus on optimization of MQWs and QBIC, as well their significant coupling for high-performance infrared detection. Meanwhile, the integration of large-scale arrays (over 1000 pixels) and pulse-coded optical computing are expected to further extend the potential applications in real-time target tracking and optical encryption communication.

## Materials and methods

### Numerical calculations

The optical response of meta-atoms was modeled via finite-difference time-domain (FDTD) simulations Lumerical FDTD Solutions. Key configurations included excitation, boundary conditions, convergence control. Specifically, the light source is *x*-polarized plane wave and illumination spanning is 6–11 μm. Periodic boundaries in *x*- and *y*-directions (periodicity: 2.5 μm), and 8-layer perfectly matched layers (PML) is adopted in *z*-direction. The dielectric properties of gold, titanium, and gallium arsenide were adopted from Palik data. Intersubband transitions were computed using the transfer matrix method (TMM) for both infinite-square-well mode and finite-square-well mode, where well width *L*_*w*_ (5 nm) and Al composition (*x* = 0.28) were tuned to achieve 8-μm absorption.

### Growth and characterization of MQWs

Epitaxial growth of the MQWs structure was performed using VEECO’s D125 Metal-Organic Chemical Vapor Deposition (MOCVD) system. The source materials used included TMGa (trimethylgallium), TMAl (trimethylaluminum), AsH_3_ (arsine), SiH_4_ (silane), and Si_2_H_6_ (disilane). The substrate selected was semi-insulating GaAs from AXT. To precisely control the n-type doping concentration within the quantum well, we designed an epitaxial structure consisting of four successive GaAs layers grown on a semi-insulating GaAs substrate. The SiH_4_ flow rates were set at 25 cc, 50 cc, 100 cc, and 150 cc for each layer, with each layer having a thickness of 500 nm. The doping concentrations of these layers were measured using electrochemical capacitance-voltage (ECV) profiling. The results, shown in Fig. S12, indicate that as the SiH_4_ flow rate increases, the measured GaAs layer concentrations correspondingly rise to 2 × 10^17^, 5 × 10^17^, 1.2 × 10^18^, and 1.7 × 10^18^ cm^–3^. This demonstrates a nearly proportional relationship between the SiH_4_ flow rate and the doping concentration. The key epitaxial growth conditions were as follows: a reaction chamber pressure of 85 mbar, growth temperatures ranging between 600 °C and 730 °C, V/III ratios from 50 to 200, and back-polished epitaxial wafers to ensure smooth and high-quality layers. A 500 nm thick GaAs layer with a silicon doping concentration of 1 × 10^18 ^cm^−2^ was grown on semi-insulating GaAs substrates to serve as the bottom contact. The active region of the quantum well consists of a 50-period alternating structure of GaAs/Al_0.28_Ga_0.72_As heterojunctions, where each period includes a 5 nm thick GaAs quantum well and a 40 nm thick barrier layer. The doping concentration in the quantum well region is 5 × 10^17 ^cm^−2^. Electrical contacts are formed by a 500-nm-bottom and 200-nm-top doped contact layer. The total thickness of the structure, which comprises the quantum well heterostructure and the two contact layers, is 2.7 μm.

The MQW structure was characterized using three different methods: photoluminescence (PL) spectrum, Fourier-transform infrared (FTIR) spectroscopy, and X-ray diffraction (XRD) patterns (see Fig. 3 in the main text). The peak of PL is measured at 816 nm, indicating that the energy level difference between the electron and hole traps in the ground state is 1.52 eV, which aligns with the design specifications. The full width at half maximum (FWHM) is recorded as 22 nm, suggesting a consistent growth structure across the 50 quantum wells. The increase in intensity observed between 840 nm and 860 nm can be attributed to intrinsic absorption caused by a 500 nm thick layer of GaAs on the material’s surface. The response wavelength was determined to be at 7.7 μm by FTIR. The good periodicity and interface quality of MQWs were characterized by XRD patterns. The numerous satellite peaks in the diffraction pattern indicates a good periodicity of the MQWs. Furthermore, the low differences between GaAs and Al_0.28_Ga_0.72_As peaks, which is less than 100 arcseconds, confirms the high crystalline quality of the heteroepitaxial layer.

### Pixelated metasurface and sample fabrication

The process flow of BIC-MQWs sample was shown in Fig. S13. A mask of SiO_2_ was used to etch down to the bottom contact layer using a mixed solution of methanol (CH_3_OH), phosphoric acid (H_3_PO_4_), hydrogen peroxide (H_2_O_2_), and water (H_2_O) in a ratio of 2:1:1:5 at 25 °C. A 300 nm thick SiO_2_ layer was then deposited through Plasma Enhanced Chemical Vapor Deposition (PECVD) using a radio frequency power of 70 W and a SiH_4_/N_2_O gas flow ratio of 1:3. After photolithographically patterning the electrode windows, a multilayer metal film of AuGeNi/Au (the thickness is 50 nm/300 nm) was deposited using magnetron sputtering. A lift-off process was employed to remove the excess metal, thereby forming low-resistance contact electrodes. Additionally, nine sets of metal metasurface arrays with asymmetric parameter gradient distributions were fabricated using EBL, and the process parameters included an acceleration voltage of 30 kV, a beam current of 10 pA and the dose of 1400 μC/cm^2^. Each set measures 160 μm × 160 μm, with a metal layer thickness of 55 nm (including a 5 nm titanium adhesion layer and a 50 nm gold layer). The gradient parameters include varying lengths of the asymmetric metal arms (from 1.8 to 1 μm) defined by δ = (0, 0.06, …, 0.44). The microscope photo of the sample was shown in Figs. S14–S15.

### Equipment and characterization

We conducted a transmittance test of the BIC metasurface using a Fourier transform infrared spectrometer (FTIR, VERTEX80, BRUKER) equipped with a microscope. A ×36 objective, a mercury−cadmium−telluride detector, a KBr beam splitter, an infrared polarizer, and a mid-wave source were used to characterize the spectra in the 6–11 μm range. All measured spectra were normalized with respect to bare samples without metasurface. The photocurrent spectra of BIC-MQW devices were characterized with the same FTIR, where the mid-wave source was first passed through the Michelson interferometer before being focused onto the sample. The collected photocurrent signals were first amplified by a low-noise current preamplifier (SR570, Stanford Research Systems Inc) before being connected with the external port of the FTIR. The optoelectrical characterization of BIC-MQWs device was conducted in a liquid nitrogen Dewar, using a current preamplifier (SR570, Stanford Research Systems Inc), a lock-in amplifier (SR865A, Stanford Research Systems Inc). During the measurement process, BIC-MQW to be tested is mounted onto the cold finger of the Dewar flask using a low-temperature adhesive. The electrode leads are routed through the Dewar’s terminal. After evacuating the system, liquid nitrogen is poured into the Dewar to achieve the required cooling, and measurements are conducted once the temperature stabilizes. The light source of an 8 μm QCL laser, which is directed onto the BIC-MQW device through a germanium (Ge) single-crystal window incorporated in the chopper and Dewar assembly, was first adjusted for power using an optical attenuator and then passed through a half-wave plate and a polarizer to modulate its polarization state. The device is biased with a current preamplifier (SR570, Stanford Research Systems Inc), and the resulting photocurrent, generated by the IR radiation, is collected from both ends of the device. Next, the linearly polarized light was modulated at a frequency of 1000 Hz using a mechanical chopper (SR542, Stanford Research Systems Inc). This current is then transmitted to a lock-in amplifier (SR865A, Stanford Research Systems Inc), where the signal is processed and recorded. The photo of measurement system saw Figs. S17–S18 and the experimental results were characterized in Figs. S19–S20.

## Supplementary information

Supplementary Information for Quasi-bound States in the Continuum Driven Photoresponse in Multiple Quantum Wells for Machine Vision

## Data Availability

The authors declare that the data supporting the conclusions of this study are available within the article and its supplement. Additional data related to the article are available from the corresponding author upon reasonable request.
