# Supplementary material for: Quasi-bound states in the continuum driven photoresponse in multiple quantum wells for machine vision
Source: Light Sci Appl. 2026 Jul 3;15:302. doi: 10.1038/s41377-026-02404-4 (PMC13328746; doi:10.1038/s41377-026-02404-4)

Supplementary Information for

**Quasi-bound States in the Continuum Driven Photoresponse in Multiple Quantum Wells for Machine Vision**

Wenjuan Zhou^a, †^, Jun Deng^a, †^, Pengying Chang^a, †^, Hongrui Dou^a^, Boyu Yang^b^, Yan Chen^b^, Peipei Chen^c^, Chen Xu^a^, Jinchao Tong^b,^ *, Jianlu Wang^b,^ *, Yiyang Xie^a,^ *, Junhao Chu^b^

*^a^ Key Laboratory of Optoelectronics Technology, Beijing University of Technology, Ministry of Education, Beijing 100124, China.*

*^b^ Institute of Optoelectronics, College of Future Information Technology, College of Integrated Circuits & Micro-Nano Electronics, Fudan University, Shanghai 200433, China.*

*^c^ Nanofabrication Laboratory, CAS Key Laboratory for Nanophotonic Materials and Devices, National Center for Nanoscience and Technology, Beijing 100190, China.*

*†These authors contributed equally to this work*

**E-mail: jctong@fudan.edu.cn; jianluwang@fudan.edu.cn; xieyiyang@bjut.edu.cn*

**The file includes:**

Supplementary Text

Figs. S1 to S34

Tables S1 to S3

Supplementary Text

1. Design of BIC metasurface

1.1 Simulated results of BIC metasurface

We simulated the wavevector |***k****_x_*|, |***k****_y_*| and the wavevector ***k*** on the *yz* plane, the monitor was positioned at the interface between the metal nanostructure and the MQWs, for the case of vertical illumination of the light source, as shown in Figs. S2 - S4.

To analyze the mechanisms, we also simulated the electric field ***E****_z_* of the structure on the *xy* plane, as shown in Fig. S5. Specifically, the distribution of energy flow, as described by the Poynting theorem, leads to the observed changes in the electric field pattern as a function of asymmetry in the structure.

With an increase in the asymmetric parameter δ, the wavevector progressively intensifies, and this pattern also applies to ***E****_z_*. This is to say, during the transition from BIC to QBIC, the leaky mode becomes more pronounced, the quantity of lateral wavevector |***k****_x_*| and |***k****_y_*| increases, and the electric field in the *z*-direction |***E****_z_*| strengthens.

1.2 Theoretical analysis of BIC metasurface

To comprehensively analyze the mechanism underlying the formation of QBIC, we selected a structure featuring a specific metasurface with an asymmetric parameter δ of 0.44. The Cartesian multipolar decomposition was employed to evaluate the scattering power in the far field of the metasurface, as illustrated in Fig. S6a. The multipole moments were derived by integrating either the charge density ρ(𝑟) or the current density 𝐽(𝑟). The scattered powers of various multipoles were then calculated by summing the contributions from dipoles located in all unit cells within the structure, expressed in Cartesian coordinates (23).

Electric dipole (ED) moment:

$$\begin{aligned} \mathbf{P}=\frac{1}{i\omega}\int\mathbf{J}d^{3}r\#\left( 1 \right) \end{aligned}$$

Magnetic dipole (MD) moment:

$$\begin{aligned} \mathbf{M}=\frac{1}{2c}\int\left( \mathbf{r}\times\mathbf{J} \right)d^{3}r\#\left( 2 \right) \end{aligned}$$

Toroidal dipole (TD) moment:

$$\begin{aligned} \mathbf{T}=\frac{1}{10c}\int\left[ \left( \mathbf{r}\cdot\mathbf{J} \right)\mathbf{r}-\mathbf{2}\boldsymbol{r}^{\mathbf{2}}\mathbf{J} \right]d^{3}r\#\left( 3 \right) \end{aligned}$$

Electric quadrupole (EQ) moment:

$$\begin{aligned} \mathbf{Q}_{\mathbf{e}}=\frac{1}{i\omega}\int\left[ r_{\alpha}J_{\beta}+r_{\beta}J_{\alpha}-\frac{2}{3}\left( \mathbf{r}\cdot\mathbf{J} \right) \right]d^{3}r\#\left( 4 \right) \end{aligned}$$

Magnetic quadrupole (MQ) moment:

$$\begin{aligned} \mathbf{Q}_{\mathbf{m}}=\frac{1}{3c}\int\left[ \left( \mathbf{r}\times\mathbf{J} \right)_{\alpha}r_{\beta}+\left( \mathbf{r}\times\mathbf{J} \right)_{\beta}r_{\alpha} \right]d^{3}r\#\left( 5 \right) \end{aligned}$$

The equations for computing the far-field scattered power corresponding to each polar moment are as follows:

Electric dipole:

$$\begin{aligned} ED=\frac{2\omega^{4}}{3c^{3}}\left| \mathbf{P} \right|^{2}\#\left( 6 \right) \end{aligned}$$

Magnetic dipole:

$$\begin{aligned} MD=\frac{2\omega^{4}}{3c^{3}}\left| \mathbf{M} \right|^{2}\#\left( 7 \right) \end{aligned}$$

Toroidal dipole:

$$\begin{aligned} TD=\frac{2\omega^{6}}{3c^{5}}\left| \mathbf{T} \right|^{2}I_{Q}^{e}\#\left( 8 \right) \end{aligned}$$

Electric quadrupole:

$$\begin{aligned} EQ=\frac{\omega^{6}}{5c^{5}}\left| Q_{e} \right|^{2}\#\left( 9 \right) \end{aligned}$$

Magnetic quadrupole:

$$\begin{aligned} MQ=\frac{\omega^{6}}{20c^{5}}\left| Q_{m} \right|^{2}\#\left( 10 \right) \end{aligned}$$

The computing formula of the total far-field scattered power is as follows:

$$\begin{aligned} I=\frac{2\omega^{4}}{3c^{3}}\left| \mathbf{P} \right|^{2}+\frac{2\omega^{4}}{3c^{3}}\left| \mathbf{M} \right|^{2}+\frac{2\omega^{6}}{3c^{5}}\left| \mathbf{T} \right|^{2}+\frac{\omega^{6}}{5c^{5}}\left| Q_{\alpha\beta} \right|^{2}+\frac{\omega^{6}}{20c^{5}}\left| M_{\alpha\beta} \right|^{2}\#\left( 11 \right) \end{aligned}$$

where c is the speed of light, $\omega$ is the frequency of the light, and $\alpha$ and $\beta$ are the *x*- and *y*-directions of the coordinate axis. The surface current distributions of the QBIC are presented in Fig. S6b. These distributions reveal the excitation of opposing currents between the two rectangular metallic arm-shaped structures, which form an electric dipole (ED). This opposing current configuration indicates a strong coupling interaction between the structures, playing a key role in the formation of the QBIC.

1.3 Simulated results of BIC metasurface on MQW structure

Similar to 1.1, we simulated and integrated the wavevector |***k_x_***|, |***k_y_***| and the electric field |***E****_z_*|^2^ on the *xy* plane at the interface (*z* = 0.2 μm) between the metal and substrate, in the middle (*z* = 0.1 μm) of the substrate, and at the origin point (*z* = 0 μm) of the active region of MQW, respectively. As shown in Fig. S7 - S8, these results clearly indicate that as the asymmetry parameter δ increases, the leaky mode becomes more pronounced, with corresponding increasing in both the lateral wavevector |***k***| and the electric field |***E****_z_*|^2^.

1.4 Simulations of the absorption inside the MQW region

We performed quantitative finite-difference time-domain (FDTD) simulations to quantify absorption enhancement and to establish a direct link between the QBIC-enhanced |***E****_z_*| field and the measured photoresponse. First, as expected from the fundamental polarization selection rules governing intersubband transitions in quantum wells ^43-45^, we verified the |***E****_z_*| field distribution within the active region for both Bare-MQW and BIC-MQW structures under normal incidence. As shown in Fig. S9a, the |***E****_z_*| field within the MQW layer is effectively zero for the Bare-MQW structure (without metasurface). This confirms that under normal incidence the polarization selection rule strictly forbids the generation of the required ***E****_z_* component, irrespective of the total electromagnetic energy present in the structure. In contrast, for the BIC-metasurface-coupled MQW structure (Fig. S9b), the QBIC resonance generates an enhanced |***E****_z_* | field within the MQW layer. This enhanced ***E****_z_* field is precisely the component that can couple to the intersubband dipole transition, providing the necessary condition for optical absorption. Then, we integrated the simulated |***E****_z_*|^2^ corresponding to the two structures, and the results are shown in Fig. S9c. For the Bare-MQW structure, the observed |***E****_z_*|^2^ is zero, whereas in the BIC-MQW structure, |***E****_z_*|^2^ increases as the asymmetry parameter δ grows. Next, the calculated optical absorption is presented in Fig. S9d. For the Bare-MQW structure, as noted in ref. 44, the selection rule of the intersubband transition dictates that the MQWs absorb the light with a *z*-component electric field, thereby the MQW-relevant absorption is effectively zero. In contrast, following the methodology established in ref. 46, the BIC-MQW structure exhibits clear MQW absorption at the designed wavelength. Crucially, the absorption magnitude increases with δ, in parallel with the δ-dependence of the |***E****_z_*|^2^ field enhancement. This direct correlation among δ, the |***E****_z_*|^2^, MQW absorption, and the measured photoresponse demonstrates that the enhanced photoresponse arises from ***E****_z_*-driven intersubband dipole transitions in the MQWs.

2. The design of MQW structure

2.1 Calculate transition energy from response wavelength

In this work, the proposed absorption wavelength of MQW is 8 µm, therefore, the photon energy is:

$$\begin{aligned} E_{\mathrm{photon}}=\frac{hc}{\lambda}=0.155 eV\#\left( 12 \right) \end{aligned}$$

Then, the transition energy from the ground state (E_1c_) to excited state (E_2c_) in the corresponding quantum well (intersubband transition, ISBT) is:

$$\begin{aligned} {\Delta E}_{c}=E_{2c}-E_{1c}=0.155 eV\#\left( 13 \right) \end{aligned}$$

2.2 Determine the bandgap energy of Al*_x_*Ga*_1-x_*As and its band offset to GaAs

For Al*_x_*Ga*_1-x_*As, the bandgap energy can be expressed as:

$$\begin{aligned} {Eg}_{AlGaAs}=1.424+1.247x, (0\leq x\leq0.45)\#\left( 14 \right) \end{aligned}$$

According to ref. (43, 52), the band offset of GaAs/Al*_x_*Ga*_1-x_*As quantum wells is adopted in a ratio of 60:40:

$$\begin{aligned} \Delta E_{c}=\left( {Eg}_{AlGaAs}-{Eg}_{GaAs} \right)\times0.6\approx0.7482x\#\left( 15 \right) \end{aligned}$$

$$\begin{aligned} \Delta E_{v}=\left( {Eg}_{AlGaAs}-{Eg}_{GaAs} \right)\times0.4\approx0.4988x\#\left( 16 \right) \end{aligned}$$

2.3 Calculate the energy levels of QWs (for infinitely high barriers approximation)

For infinitely high barriers and parabolic bands, assuming V_0_ = ∞, the wave function is zero at the boundary, as shown in Fig. S11a. The energy levels in the well are simply given by:

$$\begin{aligned} E_{i}=\frac{i^{2}\hbar^{2}\pi^{2}}{2m^{*}L^{2}}\#\left( 17 \right) \end{aligned}$$

where *L* represents the width of the quantum well, *m** denotes the effective mass within the well, *m** = 0.067 *m*_0_ for GaAs taken from ref. 52), *m*_0_ is the mass of free electrons, $\hbar$ is the reduced Planck constant, and *i* is subband subscript in the well. Then the transition energy is simply given by

$$\begin{aligned} E_{2}-E_{1}=\left( {3\hbar^{2}\pi^{2}}/{2m^{*}L^{2}} \right)\#\left( 18 \right) \end{aligned}$$

Using (13) and (18), we can get *L* ≈ 5.6 nm.

2.4 Finite-square-well correction

For the finite high barrier quantum well shown in Fig. S11b, this is fundamental to the operation of MQW-based infrared detectors and emitters. The schematic diagram in Fig. S11c illustrates the energy band diagram of the sub-bands within the quantum well structure, highlighting the energy levels involved in these intersubband absorption processes, there are$\begin{aligned} V\left( z \right)=\left\{ \begin{matrix} {\Delta E}_{c}, \left| z \right|\geq L/2 \\ 0, \left| z \right|<L/2 \end{matrix} \right.\#\left( 19 \right) \end{aligned}$

Solving the Schrödinger equation:

$$\begin{aligned} \left[ -\frac{\hbar^{2}}{2}\frac{d}{dz}\frac{1}{m^{*}}\frac{d}{dz}+V\left( z \right) \right]\varphi\left( z \right)=E\varphi\left( z \right)\#\left( 20 \right) \end{aligned}$$

Solving the equation (20), the discrete energy level *E_i_* can be obtained. However, the analytical solution necessitates solving transcendental equations, with even and odd order energy levels corresponding to different equations, making this a cumbersome task. In practical device design, numerical methods, such as the Transfer Matrix Method (TMM)) (*43*, *53*), is commonly employed due to its efficiency by iteration. The process is as follows:

1. Initial guess: Assuming an initial Al component *x* (i.e. *x* = 0.3), according to equations (15) and (16), $\Delta E_{c}=0.22446 eV$, $\Delta E_{v}=0.14964 eV$;
2. Assuming the width of the quantum well: Assuming *L* = 5.6 nm that is obtained from infinite barrier assumption;
3. Numerical solution: Use TMM (43, 53) to calculate the energy levels E_2c_ and E_1c_ under the (*x*, *L*) parameter;
4. Check transition energy: Calculate ${\Delta E}_{c}=E_{2c}-E_{1c}$;
   1. if ${\Delta E}_{c}>0.155 eV$, this suggests that the well is either too narrow or the barrier is too high. In such cases, it is necessary to either increase *L* or decrease *x* to achieve the desired performance.
   2. if ${\Delta E}_{c}<0.155 eV$, this indicates that the well is either too wide or the barrier is too low. In this situation, it is essential to reduce *L* or increase *x* to optimize the MQW's performance.
5. Iterative optimization: repeating steps 2 - 4 while continuously adjusting *x* and *L* until ${\Delta E}_{c}=0.155 eV$.

Finally, we obtain $L\approx5 nm$, $x\approx0.28$, E_1c_ ≈ 0.045 eV, E_2c_ ≈ 0.200 eV and E_1v_ ≈ 0.008 eV. E_interband_ = ${Eg}_{GaAs}+E_{1c}+E_{1v}$ $\approx$1.477 eV. To decouple and minimize interband tunneling between adjacent subbands, thereby balancing the challenges posed by dark current and bias voltage, the barrier thickness is set to 40 nm.

3. Multifunctional Photoresponse for Hardware Implementation of Machine Vision

3.1 Image Preprocessing of Contrast Enhancement using Nonlinear Photoresponse

The Fig. S21 shows the hardware implementation of image contrast enhancement by BIC-MQWs. The measured data as a function of QBIC asymmetry δ exhibits a nonlinear relationship, where the input (asymmetry δ) and output signals (photocurrent) were normalized. The δ ranging between 0 and 0.44 is linearly normalized to [0,1] interval, and the corresponding photoresponse is also linearly normalized to [0,1].

The normalization of δ can be expressed as

$$\begin{aligned} Normalized \delta=\frac{\delta-\delta_{min}}{\delta_{max}-\delta_{min}}\#\left( 21 \right) \end{aligned}$$

where *δ_min_* and *δ_max_* are the minimum and maximum *δ* values of designed metasurface.

The normalization of photoresponse expressed in photocurrent can be expressed as

$$\begin{aligned} Normalized photoresponse=\frac{I-I_{min}}{I_{max}-I_{min}}\#\left( 22 \right) \end{aligned}$$

where *I_min_* and *I_max_* are the minimum and maximum photocurrent values of BIC-MQWs.

The obvious nonlinear concave curve manifests the possible functionality in image contrast enhancement. When the input images are converted to the asymmetry δ, the pattern pixels with larger δ were enhanced with large photocurrent, while the pattern pixels with smaller δ were conversely degraded with small photocurrent. These changes result in the enhanced pixel signal ratio between the body pattern pixels and the background pixels in terms of the photocurrents through photoelectrical conversion. The unique geometrical asymmetry-dependent photocurrents observed in the QBIC-MQW device suggest the capability of in-sensor image preprocessing, including the image contrast enhancement and noise reduction.

Note that the images introduced to an optical pattern recognition system are normally an amplitude representation of the original intensity images. The input image-to-δ mapping is a pixel-wise, pre-calibrated one-to-one encoding process, so that each input image pixel exactly matches one metasurface unit cell with targeted δ. This mapping process can be implemented by off-chip and on-chip strategy. The off-chip one is achieved by using a self-developed Python/MATLAB script to perform independent mapping calculation for each pixel’s grayscale value obtained from intensity value, and obtained the target δ value for the corresponding metasurface pixel. The on-chip one is when input images are encoded into light intensity, input signals are transformed into electrical signal via photodectector, and subsequently the electrical signal is encoded into asymmetry parameter δ through control circuit.

Additionally, the work principle of contrast enhancement based on BIC-MQW’s photocurrent-δ characteristics is explained in detail. As shown in Fig. S22a, we discuss the linear response (green) without contrast enhancement and the nonlinear concave response (red) with contrast enhancement. Note that the input asymmetry parameter δ and the ouput photoresponse signal are both normalization to range [0,1]. For input image, the body pattern pixels are mapped into high values of δ, while background pixels are low values, both of which within range [0,1]. Next, we perform contrast enhancement estimation by considering four points: A1 and A2 corresponding to background pixels, B1 and B2 corresponding to body pattern pixels. The estimated results are listed in Fig. S22b. For contrast between A1 and B1, the input contrast ratio of δ is 0.75/0.25 = 3, and the output contrast ratio of photoresponse after preprocessing is obtained to be 3 using linear photoresponse-δ, while it increases up to 78 using nonlinear concave photoresponse-δ, leading to an enhancement by 26 times. For contrast between A2 and B2, the input contrast ratio of δ is 0.8/0.4 = 2, and the output contrast ratio of photoresponse after preprocessing is obtained to be 2 using linear photoresponse-δ, while it increases up to 8 using nonlinear concave photoresponse-δ, leading to an enhancement by 4 times with BIC-MQW preprocessing. These detailed calculations explain the principles of contrast enhancement using nonlinear concave photoresponse-δ obtained from BIC-MQW system.

3.2 In-Sensor MAC Operation using Linear Photoresponse

Having demonstrated the switchable and linear photoresponsivity of BIC-MQW dependent on different bias voltage amplitude and polarity, it is of interest to implement the multiply-accumulation (MAC) operation using the BIC-MQWs, as shown in Fig. S23. When mapping the input image to the BIC-MQWs, the pixel value of +1 and -1 are represented by applying and removing illumination stimuli to the BIC-MQWs. The kernel weights were mapped to the photoresponsivity of the BIC-MQWs, which can be directly modulated by external bias voltage. The dot-product operation between input image and kernel weight was obtained through a summation of photocurrent produced by 3 × 3 = 9 BIC-MQWs through MAC operation, which is a fundamental operation in realizing the in-sensor computing architectures for the neuromorphic visual system. For an image sensor array that consists of *N* pixels arranged in a 2D array, i.e., *N* = *H* × *W*, where *H* and *W* are the height and width of the array. Each BIC-MQWs positioned at each pixel is operated under designed bias and under optical illumination, outputting a photocurrent of *I_mn_* = *R_mn_P_mn_* (*m*=1,2,…,*H*, and *n* = 1, 2, …, *W*), where *R_mn_* is the photoresponsivity of the pixel, *P_mn_* is the corresponding input optical power. Summing all photocurrents produced by each BIC-MQWs based on the Kirchhoff’s law, the total output current is described as

$$\begin{aligned} I=\sum_{m=1}^{H} \sum_{n=1}^{W} R_{mn}P_{mn}\#\left( 23 \right) \end{aligned}$$

achieving the MAC operation.

4. Image Preprocessing and Neural Network

4.1 Generation of Original Data for Four Types of Image Patterns

For the letters ‘B’, ‘J’, ‘U’, and ‘T’, we generated corresponding standard images with 7×7 pixel array, and then derived their variants, and finally added background noise to each image. Taking the letter ‘J’ as an example, the body pattern pixel value is 1, and the background pixel value is 0. One example of its variants can be obtained by randomly selecting one body pattern pixel with a value of 1 and set its value to 0 based on the standard ‘J’ image data. Subsequently, the background pixel with a value of 0 were randomly assigned the values between 0 and 0.5 following a uniform distribution. After deriving the variant patterns, the noise signal was set to Gaussian noise with a mean of 0 and a standard deviation of 0.05, to simulate the non-ideal factors in real image. The pixel values were then normalized. The above-mentioned image generation process is shown in Fig. S24a. Similarly, the same operation was applied to the other three letters. For each letter, 120 images were generated. Therefore, we obtained a total of 480 original images, with half used for training and half for testing. Using the relationship curve between the normalized asymmetry δ and the normalized photocurrent signal shown in Fig. S24b, a dataset with contrast-enhanced images is obtained. These two datasets of the original images and the contrast-enhance images were fed into the artificial neural network for image recognition, respectively.

4.2 Different running cycles with different noise signals

Fig. S25 show the comparisons of recognition accuracy in artificial visual system with and without BIC-MQW preprocessing at different 8 simulation running cycles. The background noise signals are generated randomly in 8 running cycles. For the image database with different background noise, the recognition rates with BIC-MQW preprocessing are always higher than that without BIC-MQW preprocessing, confirming the repeatability of the results.

4.3 Different running cycles with different noise signals

Supplementary Table S3 Epoch number for the image recognition tasks with and without BIC-MQW-based preprocessing. The improvement rate in epoch number with the inclusion of BIC-MQW are also listed in the table.

4.4 Simulated wavelength-dependent recognition performance

We elaborate on the wavelength-dependent recognition performance from two aspects: simulation analysis of the |***E****_z_*|^2^ and calculation of the recognition accuracy.

Simulated integral of the |***E****_z_*|^2^ from 6 μm to 10 μm: To evaluate the wavelength dependence of the field enhancement, we performed FDTD simulations of the |***E****_z_*|^2^ within the active region for wavelengths from 6 μm to 10 μm in 0.5 μm steps. The results are presented in Fig. S10. The integral of the |***E****_z_*|^2^ exhibits pronounced nonlinear variation in the 7 - 8 μm range, which includes the designed resonance. This behavior confirms that the device is optimized for 8 μm operation but retains useful enhancement over approximately a 1 μm bandwidth, matching the FTIR-measured MQW absorption width. These simulation results corroborate that the contrast enhancement and subsequent machine-learning performance are effective within the target spectral window, and degrade as the wavelength deviates from the resonance peak, which is verified in following Fig. S26.

Wavelength-dependent recognition accuracy: Fig. S26 show the simulated wavelength-dependent recognition performance based on the QBIC-mediated |***E****_z_*|^2^ response in Fig. S10 from 6 μm to10 μm, including comparison of images before and after BIC-MQW-based preprocessing and corresponding recognition accuracy during training epochs with and without BIC-MQW-based preprocessing. From Fig. S26a with wavelength 6μm, the main body pixel values and the background pixel values are reversed after BIC-MQW-based processing. From Fig. S26b, |***E****_z_*|^2^-δ relationship shows convex curve, and thus the image contrast after preprocessing is reduced and thereby the recognition accuracy and training efficiency is degraded with preprocessing. From Fig. R26c, d and e with wavelength 7, 7.5 and 8 μm, |***E****_z_*|^2^-δ relationship shows concave curve, resulting that the image contrast after preprocessing is enhanced and thereby the recognition accuracy and training efficiency is improved. From Fig. S26f with wavelength 8.5 μm, |***E****_z_*|^2^-δ relationship begins to deviate from concave curve at large δ values, and thus performance improvement is significantly reduced. From Fig. S26g, h, and i with longer wavelength, |***E****_z_*|^2^-δ relationship severely deviates from concave curve, image contrast after preprocessing is severely degraded, and the recognition accuracy and training efficiency with preprocessing is worse than that without preprocessing. These simulation results confirm that the contrast enhancement and subsequent machine-learning performance are effective within the target spectral window (7 - 8 μm range), and degrade as the wavelength deviates from the resonance peak.

4.5 Simulated SNR-dependent recognition performance

To verify robustness against noise, the signal-noise ratio (SNR)-dependent recognition tasks is performed by considering two configurations of noise that is induced to datasets. As stated in Supplementary 4.1, the dataset consisting of letters ‘B’, ‘J’, ‘U’ and ‘J’ is generated by three steps: (1) Variants of each letter can be obtained by randomly selecting one body pattern pixel with a value of 1 and set its value to 0 based on the standard image data; (2) The background pixel with a value of 0 were randomly assigned the values between 0 and 0.5 following a uniform distribution; (3) After deriving the variant patterns, the noise signal was set to Gaussian noise with a mean of 0 and a standard deviation of 0.05. The two configurations of noise for SNR-dependent recognition study are related to steps (2) and (3) respectively. To fairly compare the recognition accuracy, the image data before adding any noise by step (2) & (3) keeps unchanged in all following simulations, of which the random seed for step (1) is the same with that in first subfigure of Fig. S24.

For the first configuration of noise related to step (2), the background pixels with a value of 0 were randomly assigned following a uniform distribution U(a_U_, b_U_), where a_U_ represent the lowest value of background pixel value, and b_U_ represent the highest value of background pixel value. The case of a_U_ = 0 and b_U_ = 0.5 is considered in manuscript. Herein, different values of b_U_ between 0 and 1 are adopted, meaning different degree of background pixel noise. Fig. S27a-27k show the simulation results obtained by different b_U_ values, including the comparison of images including letters ‘B’, ‘J’, ‘U’ and ‘J’ before and after BIC-MQW-based preprocessing, and corresponding recognition accuracy during training epochs with and without BIC-MQW-based preprocessing. Fig. S27l summarize the recognition accuracy as a function of b_U_, showing that BIC-MQW-based preprocessing is beneficial for improving the recognition accuracy especially in high noise cases. Fig. S27m gives the number of training epoch to get accuracy of 80% as a function of b_U_, where BIC-MQW-based preprocessing significantly reduces the training epoch and improves the training efficiency.

For the second configuration of noise related to step (3), both main body pixels and background pixels were added by a Gaussian noise N(μ, σ^2^), where μ is mean value and σ is standard deviation. The case of μ = 0 and σ = 0.05 is considered in manuscript. Herein, different values of σ ranges between 0 and 1 are explored, as shown in Fig. S28a-28i with σ = 0, 0.05, 0.1, 0.3, 0.5, 0.7, 0.9, 0.95, and 1. Similarly, as summarized in Fig. R18j and R18k, as noise of input image increases (i.e. σ increases), the effectiveness of BIC-MQW-based preprocessing on both recognition accuracy and training efficiency is more pronounced, verifying the robustness of BIC-MQW system against noise.

In brief, the BIC-MQW system show strong robustness against noise.

5. Pattern Classification

5.1 Construction of a Dataset for different Letter Patterns

As shown in Fig. S29, we constructed 17 different noise patterns derived from the pixel arrays of the standard letters ‘F’ and ‘D’ (including the standard patterns themselves, making a total of 17 distinct patterns). These patterns were created by randomly selecting 0, 1, or 2 pixels with a logical value of +1 from the standard letter pixel arrays and changing their logical values to -1. To ensure reproducibility of the results, we used a random seed for generating these derivative pixel arrays. For each of the letters ‘F’ and ‘D’, we generated 25 4 × 4 pixel array data using these 17 different patterns, resulting in a total of 50 4 × 4 pixel array data (25 for ‘F’ and 25 for ‘D’).

5.2 Single-Layer Perception for Binary Pattern Classification

As shown in Fig. S30, a 4 × 4 pixel array representing the letter ‘F’ is fed into a single-layer perceptron model as a column vector. The pixel values are binarized, and the input neurons receive the 16 pixel values, each connected to the output neuron with a weight $w_{i}\left( i=1,2,\ldots,16 \right)$. There is also a fixed bias input $x_{0}$ with a logical value of +1, connected to the output neuron with a weight $w_{0}$. The inputs $x_{1-16}$ and the bias $x_{0}$ are combined with their respective weights $w_{i}\left( i=0,1,\ldots,16 \right)$ through a weighted sum and a sign function operation: $y=sgn[\sum_{i=0}^{16} w_{i}x_{i}]$. The output y is then used to determine the category of the letter. The pixel arrays for the standard patterns of the letters ‘F’ and ‘D’ are shown in Fig. S30C, with their respective label values being +1 and -1.

The *ex-situ* training process is performed as follows. We initialize the weights $w_{i}\left( i=0,1,\ldots,16 \right)$ to 0. During one training epoch, the 50 sets of pixel array data are sequentially fed into the single-layer perceptron. The weights are adjusted using the perceptron learning rule, as shown in equation (61):

$$\begin{aligned} \Delta w_{i}=\alpha{x_{i}}^{\left( train \right)}\left( {y_{predict}}^{\left( train \right)}-{y_{target}}^{\left( train \right)} \right)\#\left( 24 \right) \end{aligned}$$

here, $\alpha$ is the learning rate during training, ${x_{i}}^{(train)}$ is the $i$-th input pixel value for a single pixel array data during training on the training dataset, and ${y_{predict}}^{(train)}$ and ${y_{target}}^{(train)}$ are the predicted result and target value for a single pixel array data, respectively. After several iterations of training, testing on the training set achieved a recognition accuracy rate of 100%. The simulation demonstrates that training this single-layer perceptron can effectively separate the two input patterns. For these fixed 50 sets of data, we obtained a unified set of weights $w_{i}\left( i=0,1,\ldots,16 \right)$, which are [-1, 1, -1, -1; 3, -1, 1, 1; -3, 1, 3, 3; -1, -1, -3, -3;]. Clearly, the weights that allow successful classification only require two strength values with positive and negative states to separate these input patterns, which provides feasibility for hardware implementation.

5.3 Higher-resolution input patterns mapped onto limited sensor array

In Supplementary Text 3.2 and Fig. S23, a MAC operation based on 3 × 3 BIC-MQW array is illustrated, as the input figure of letter ‘T’ is represented by 3 × 3 pixels. In fact, the pattern classification in Fig. 5e-g is performed for letters ‘F’ and ‘D’ with 4 × 4 pixels. There is a mismatch of array size between fabricated sensor array (M × M with M = 3) and images pixels (N × N with N = 4) for classification application. To perform the hardware experiment of pattern classification, we discuss the M ≥ N and M < N case, respectively. As shown in Fig. S31a and S31b, for M ≥ N case, the image can be mapped to N × N devices of overall M × M devices in sensor array, and the output current obtained through the MAC operation of N × N parallelly connected BIC-MQWs are used to determine the pattern type. In contrast, as shown in Fig. S31c, for M < N case, the N × N image pixels have to be divided to Y (= round(M/N)) parts, and the final output current is summed over each output current of MAC operation of Y parts. Due to limited sensor pixels, the 16 pixels of input image are divided into 9 pixels and 7 pixels, and the resultant currents are summed one by one.

6. Applications in edge detection

To further demonstrate the applications of QBIC-MQW devices in machine vision systems, we performed simulations on image edge detection via convolution neural network (CNN), as shown in Fig. S32. Fig. S32a shows the schematic illustration of edge detection process. A 20 × 20 image showing a diamond shape was adopted as the input image, whose pixel values of black and white were defined as 1 and 0, respectively. When mapping the input image to the QBIC-MQW devices, the pixel value of 1 and 0 are represented by applying and removing illumination stimuli to the QBIC-MQW devices. During the convolution process in the edge detection, 3 × 3 kernels were sliding over the input image with a stride of 1. Subsequently, the input image was transformed into 18 × 18 feature maps. As shown in Fig. S32b, herein we adopt two basic Sobel kernels, where the kernel 1 collects the gradient in *x* direction (*G_x_*) and the kernel 2 collects the gradient in *y* direction (*G_y_*), respectively. The kernel weights were mapped to the photoresponsivity of the QBIC-MQW devices, which is modulated by the incident angle of light shed on the QBIC-MQW devices. The dot-product operation between each sub-image and kernel was obtained through a summation of photoelectrical currents produced by 3 × 3 = 9 QBIC-MQW devices after the convolution process. The simulation results are demonstrated in Fig. S32c-32f. The output current *I_x_* and *I_y_* corresponding to kernel 1 and 2 captures the *G_x_* and *G_y_* features, respectively. These two sets of output currents can then be merged together to find the absolute magnitude of the gradient at each pixel point. The current values are further normalized and binarized to obtain the final output image, clearly detecting the edge between the diamond and the background.

7. Discussion on the broadending of the spectral intensity range and the applications

Intensity enhancement: The observed intensity increase with δ originates from the physics of symmetry-protected BICs. At δ = 0, the structure is perfectly symmetric, corresponding to an ideal BIC but zero coupling to the far field, hence the clear spectral feature cannot be observed. As δ increases, turning the BIC into a QBIC, symmetry breaking enables coupling to radiation. Intersubband transitions in MQWs scale with the integral of |***E****_z_*|^2^ over the active region, so the enhanced |***E****_z_*|^2^ directly translates to higher absorption and improved photoresponse (as shown in Figs. 2 and 4). Consequently, the intensity exhibits an increase with δ.

Spectral broadening: The QBICs withpout the sharp resonant lineshape in the curves could be attributed to two factors that wavelength-dependent material losses and material-dependent characteristics.

Wavelength-dependent material losses: The quasi-BIC resonance lineshape is governed by the quality factor (Q), which results from a balance of radiative and non-radiative losses. In our hybrid metal-multilayer structure at infrared wavelengths, material absorption plays a dominant role in broadening the resonance. As shown in Fig. S33, the imaginary part of the metallic components increases at longer wavelengths. This leads to a higher material absorption loss, a lower Q-factor, and a broader resonance linewidth. To verify this inference, we achieved BIC-like characteristics in the shorter-wavelength region (~1.3 μm) by adjusting the geometric dimensions within the same material system, as shown in Fig. S34. A direct comparison of the Q factors at λ = 1.3 μm and λ = 7.7 μm reveals that the shorter-wavelength mode exhibits a higher Q and a narrower resonance linewidth, as illustrated in Fig. S1. This phenomenon indicates that quasi-BICs do not exhibit sharp resonant lineshape in the curves, likely due to wavelength-dependent losses that broaden the resonances.

Material-dependent characteristics: Related studies show a pronounced difference in resonance lineshapes and Q-factors between dielectric and metallic (or hybrid) BIC systems. All-dielectric metasurfaces can support ultra-narrow, high-Q resonances due to low intrinsic losses in the infrared. In contrast, metallic metasurfaces suffer from Ohmic losses, which broaden the resonance and reduce the Q-factor. According to representative study^48^, dielectric-dominated quasi-BIC modes show narrow linewidths and high Q-factors, while metal-dominated modes display broader resonances due to plasmonic losses. Our structure, which includes metallic components to enhance field confinement, inherently experiences these broadening effects.

Applications: The simultaneous intensity enhancement and spectral broadening with increasing δ offer opportunities for device applications. (i) Machine vision. Increased |***E****_z_*|^2^ at larger δ benefits on-chip image processing, because higher absorption generates stronger photoresponse would improve SNR. And enhanced contrast in the nonlinear response regime enables more effective image preprocessing (contrast enhancement, edge detection). (ii) Broadband spectroscopic applications. Arrays of metasurfaces with varying δ could achieve broadband coverage, combining narrow resonances (small δ, high Q) for high-resolution sensing with broader resonances (larger δ, lower Q) for high-sensitivity detection over a wider wavelength range. (iii) Multifunctional operation. Tuning both resonance strength and linewidth via δ enables devices that switch between high-resolution (small δ) and high-sensitivity (larger δ) modes, adaptable to diverse measurement requirements.

**
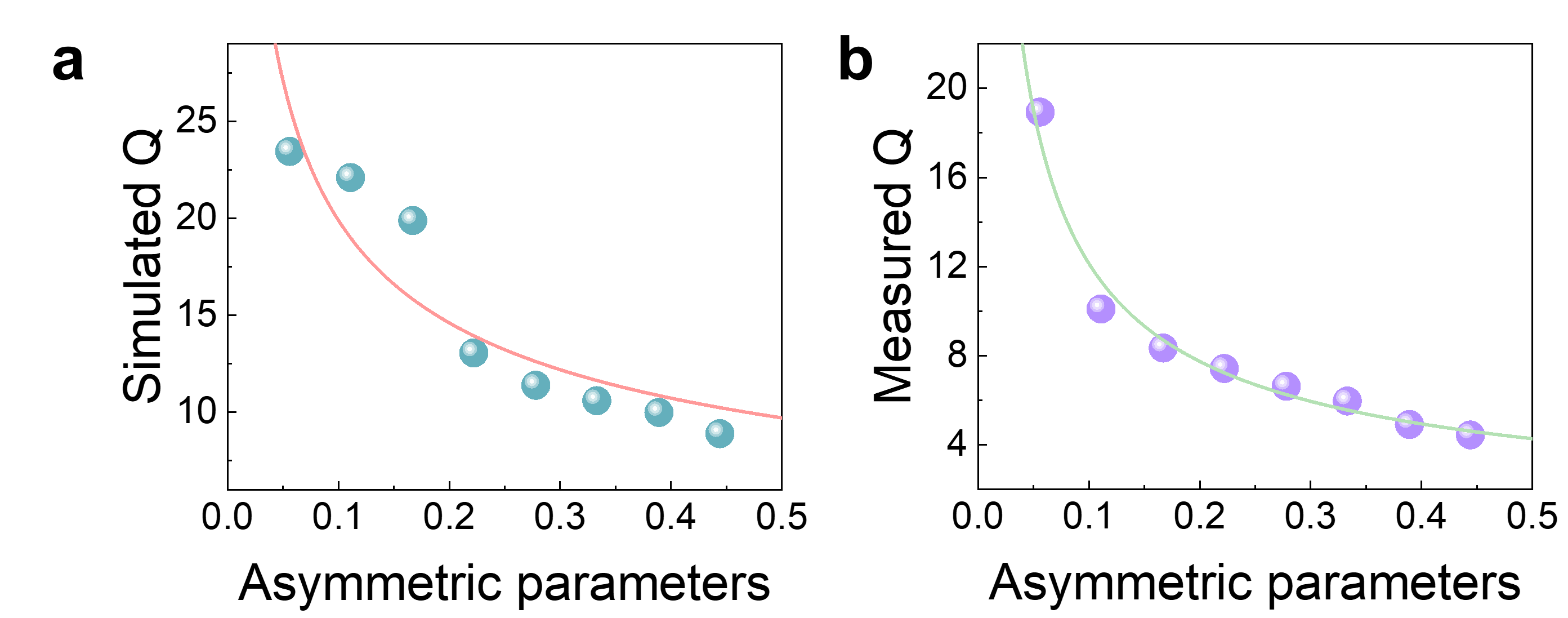
**

Fig. S1.

Comparison of simulated (a) and experimental (b) Q factors.


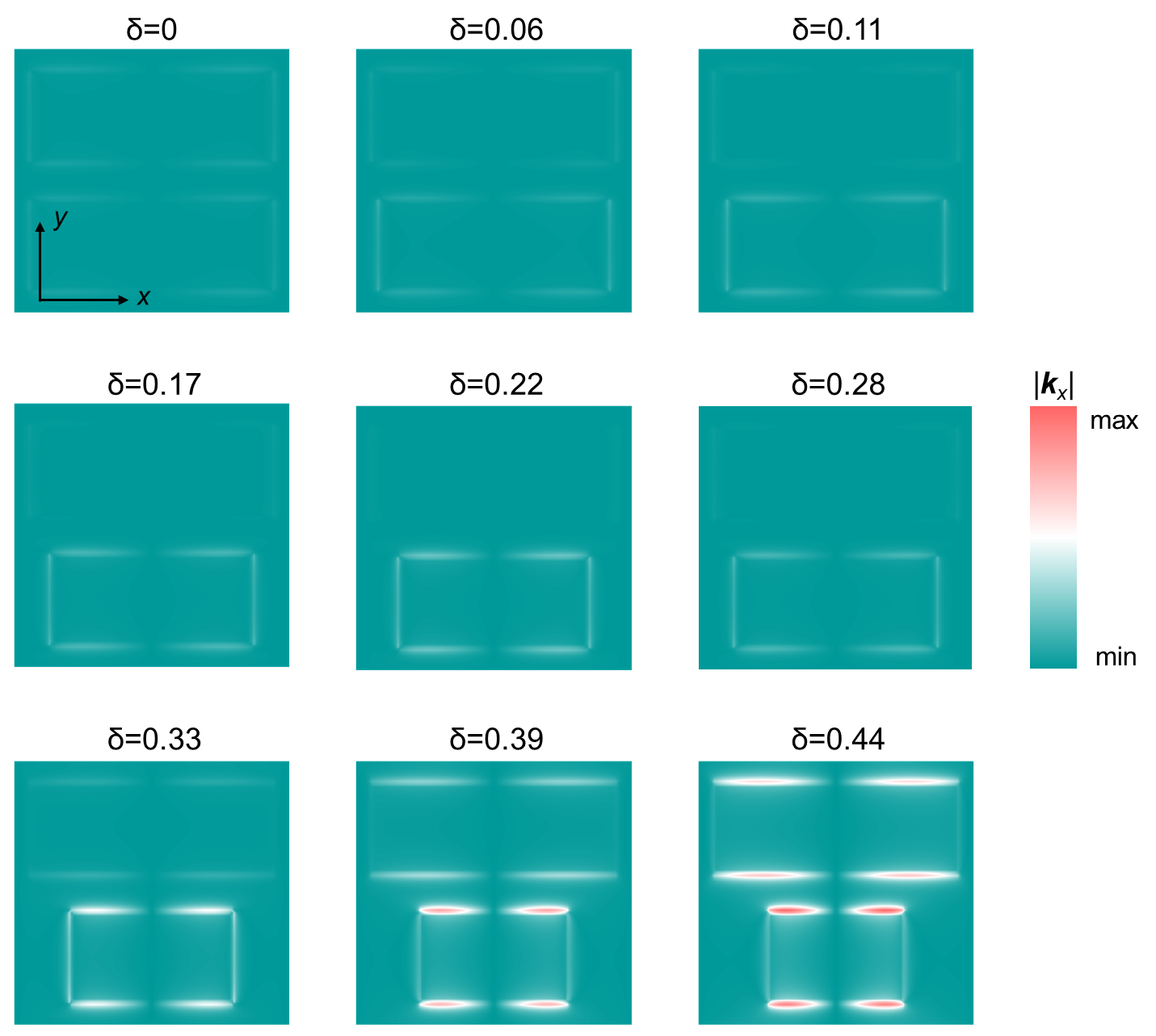


Fig. S2.

The simulated mapping relationship of the wavevector |***k****_x_*| varies as a function of δ, and the monitor positioned at the interface between the metal and the substrate.


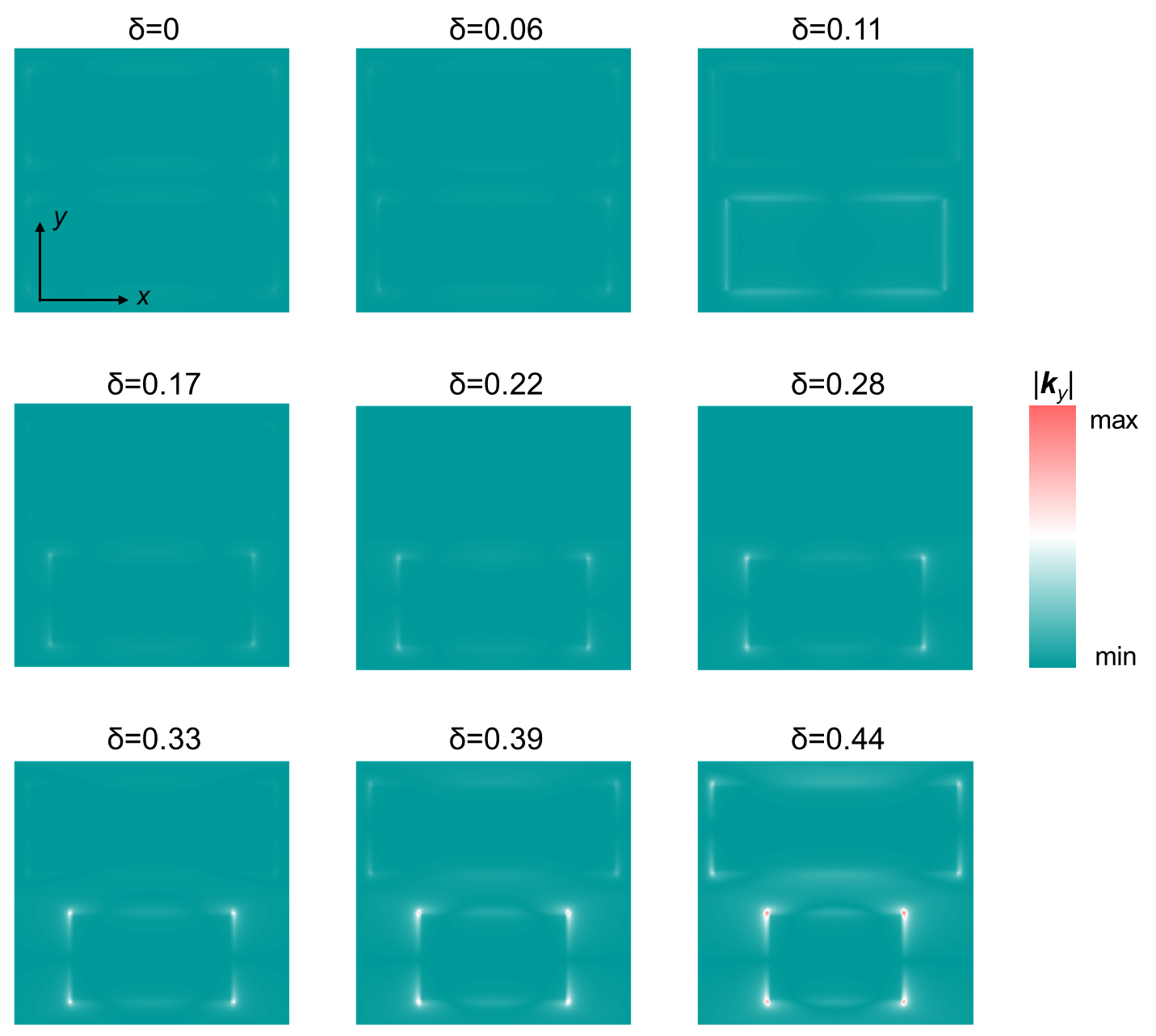


Fig. S3.

The simulated mapping relationship of the wavevector |***k****_y_*| varies as a function of δ, and the monitor positioned at the interface between the metal and the substrate.


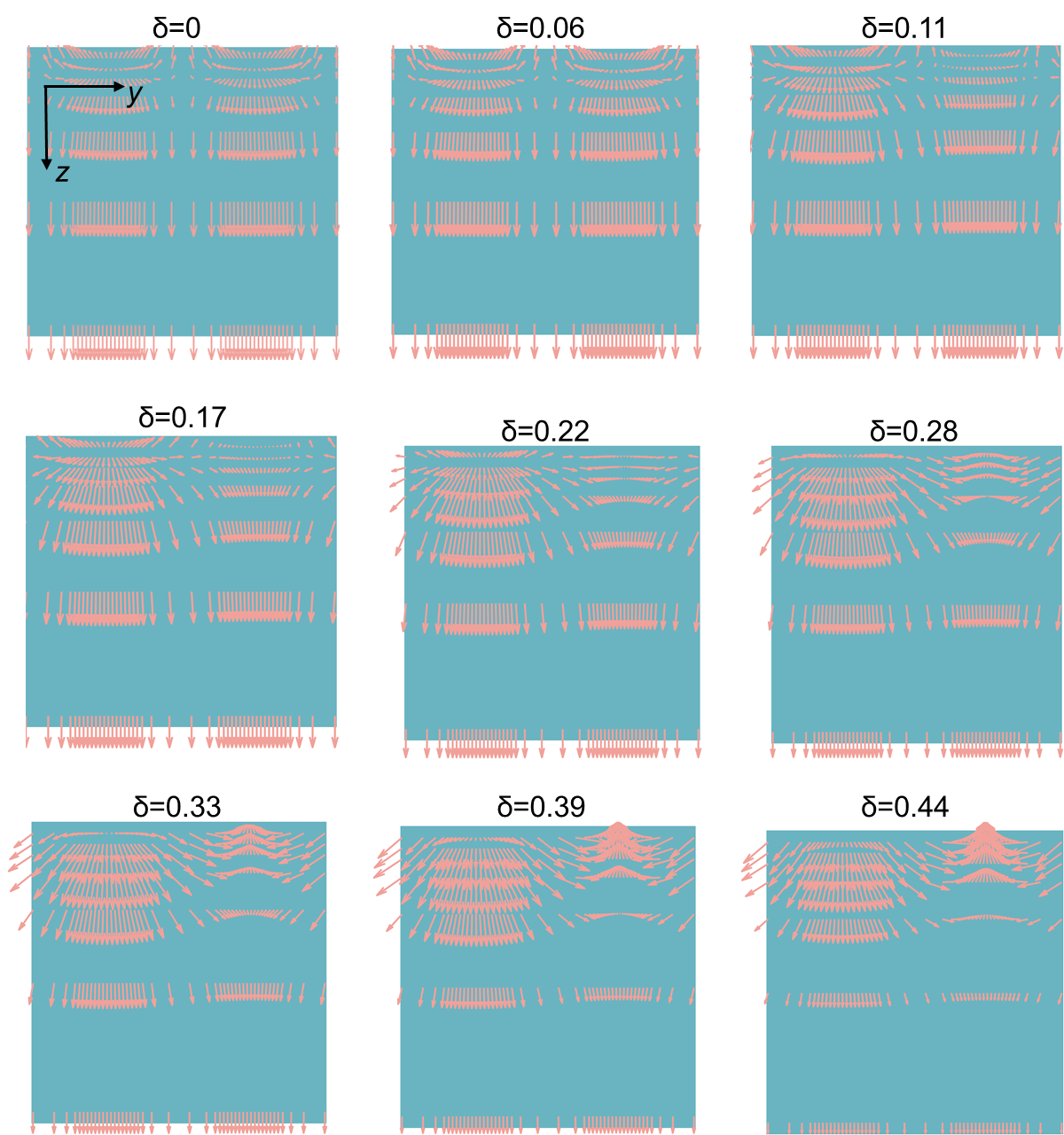


Fig. S4.

Simulated results of Poynting vector ***k*** on *yz* plane with different δ.


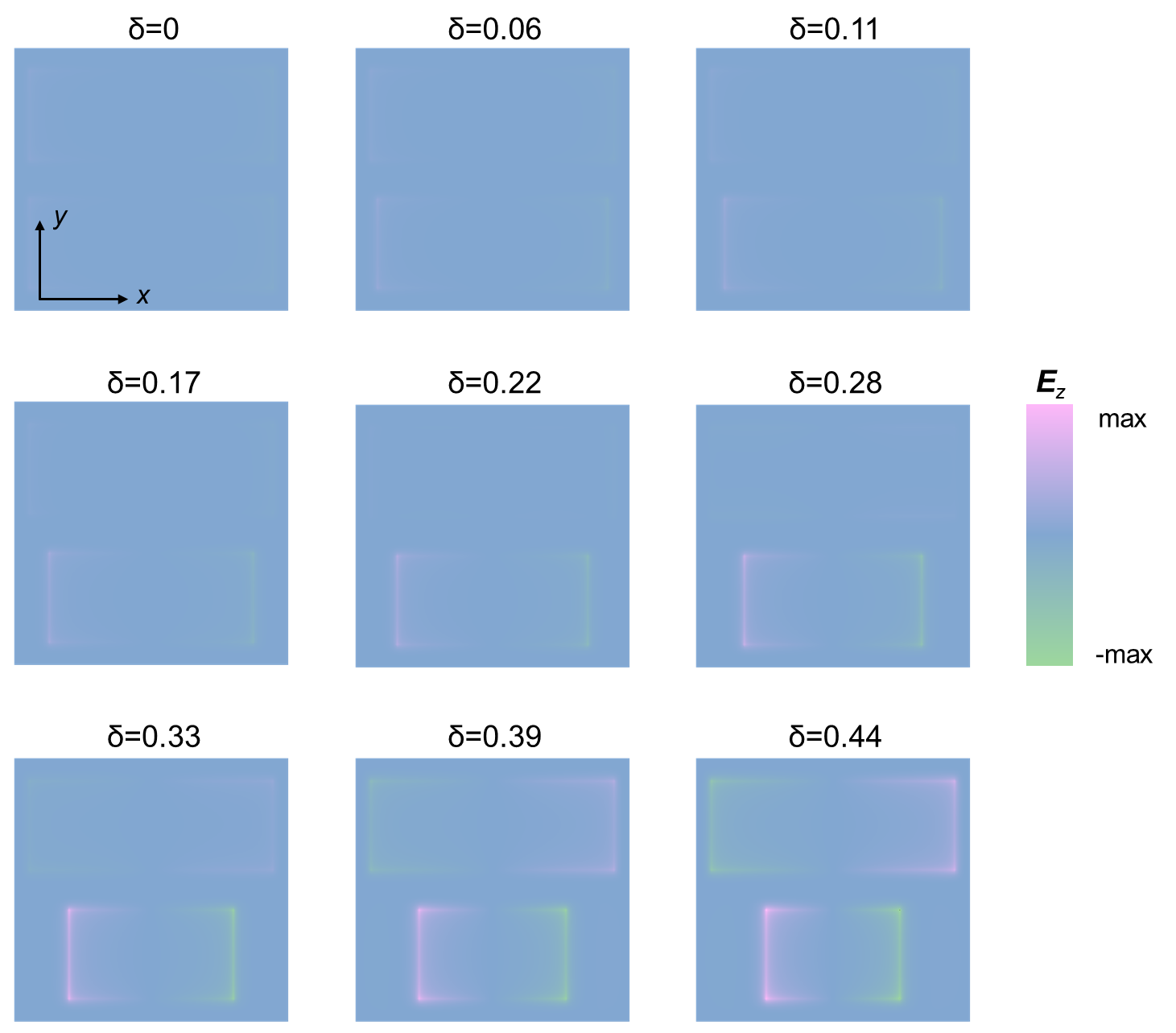


Fig. S5.

The simulated mapping relationship of the electric field component ***E****_z_* varies as a function of δ, and the monitor positioned at the interface between the metal and the substrate.


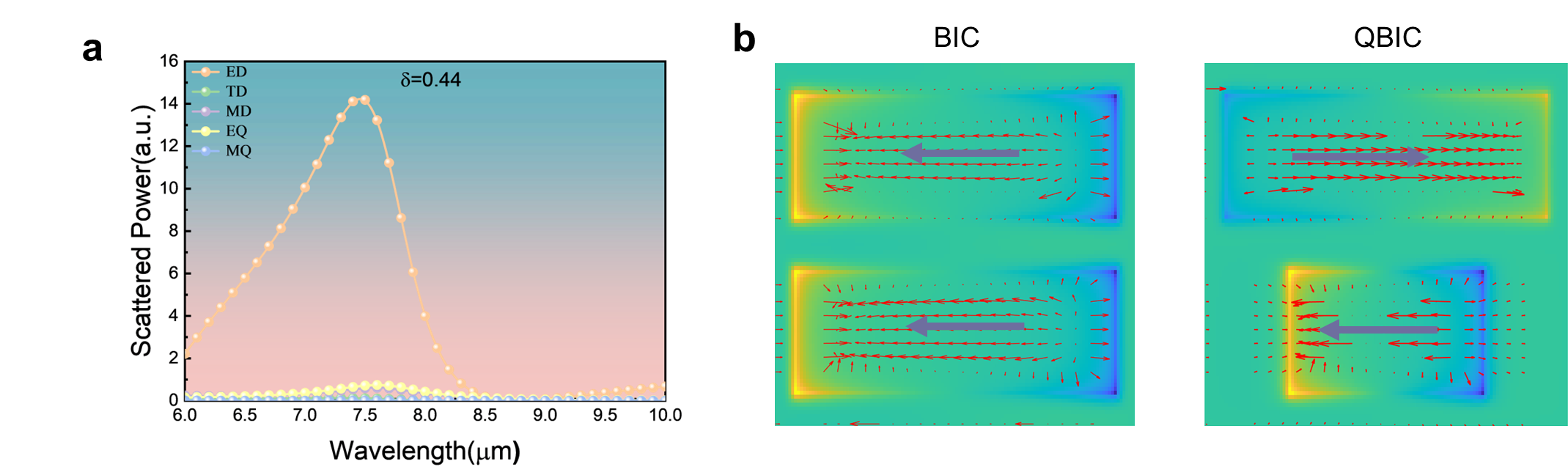


Fig. S6.

Theoretical analysis of BIC metasurface based on Cartesian multipolar decomposition. (a) The far-field scattering power of the QBIC metasurface (δ = 0.44), including the components of ED, TD, MD, EQ and MQ respectively. (b) The surface current distribution in the *xy* plane under QBIC and BIC mode.


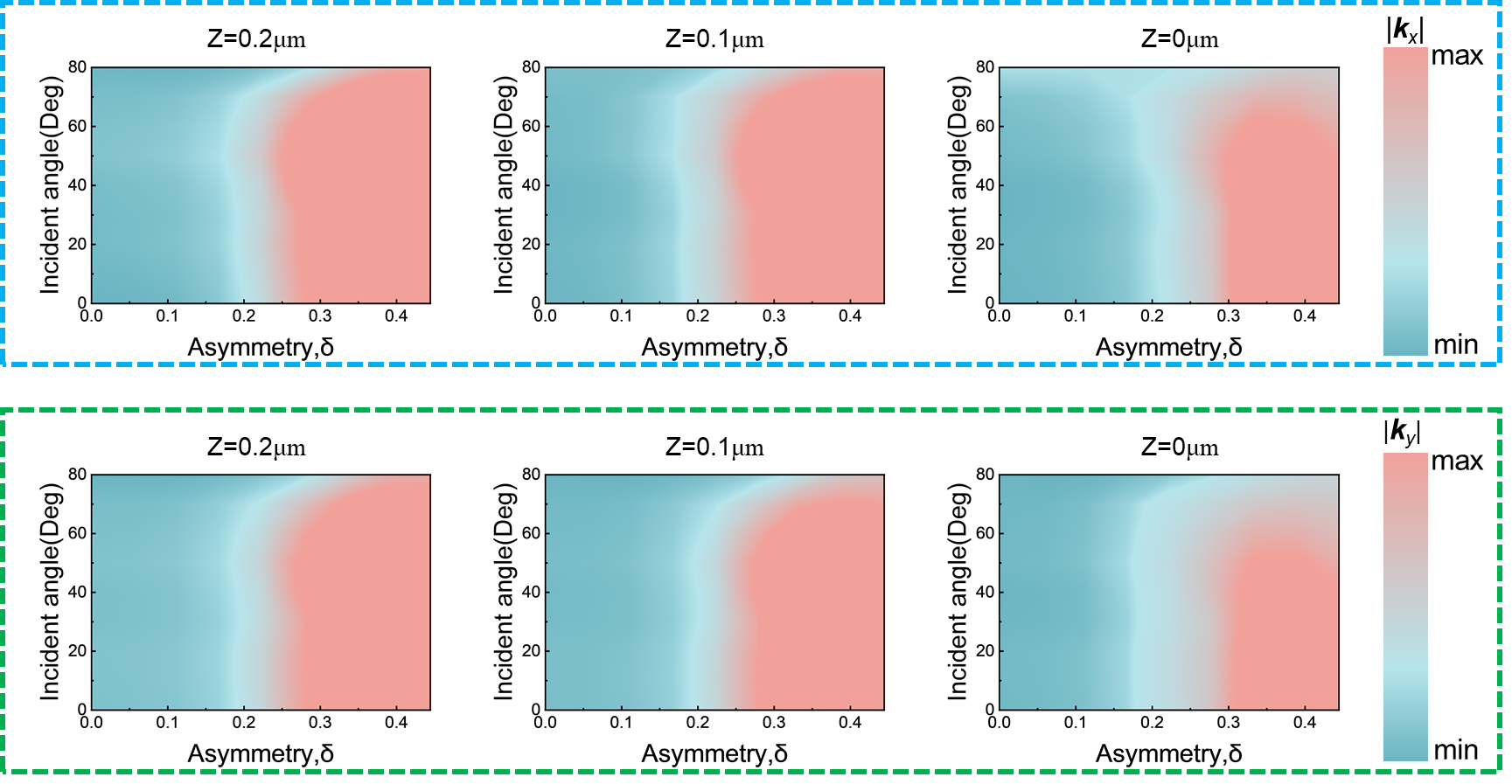


Fig. S7.

The integral of wavevector |***k_x_***| and |***k_y_***| on the *xy* plane at the interface (z = 0.2 μm) between the metal and substrate, in the middle (z = 0.1 μm) of the top contact of MQW, and at the origin point (z = 0 μm) of the active region of MQW, respectively.

**
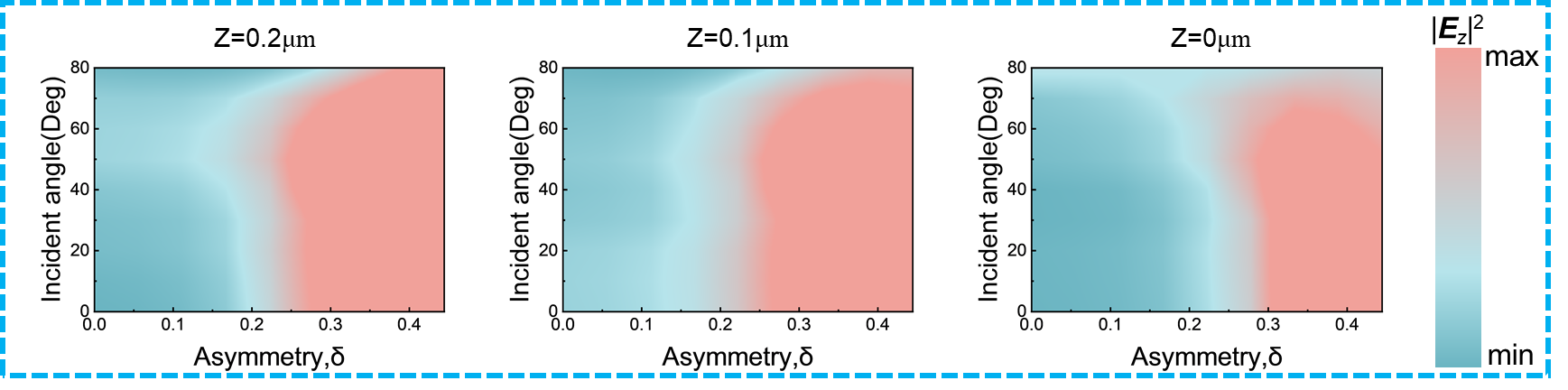
**

Fig. S8.

The integral of the electric field |***E*_z_**|^2^ on the *xy* plane at the interface (z=0.2 μm) between the metal and substrate, in the middle (z=0.1 μm) of the top contact of MQW, and at the origin point (z=0 μm) of the active region of MQW, respectively.

**
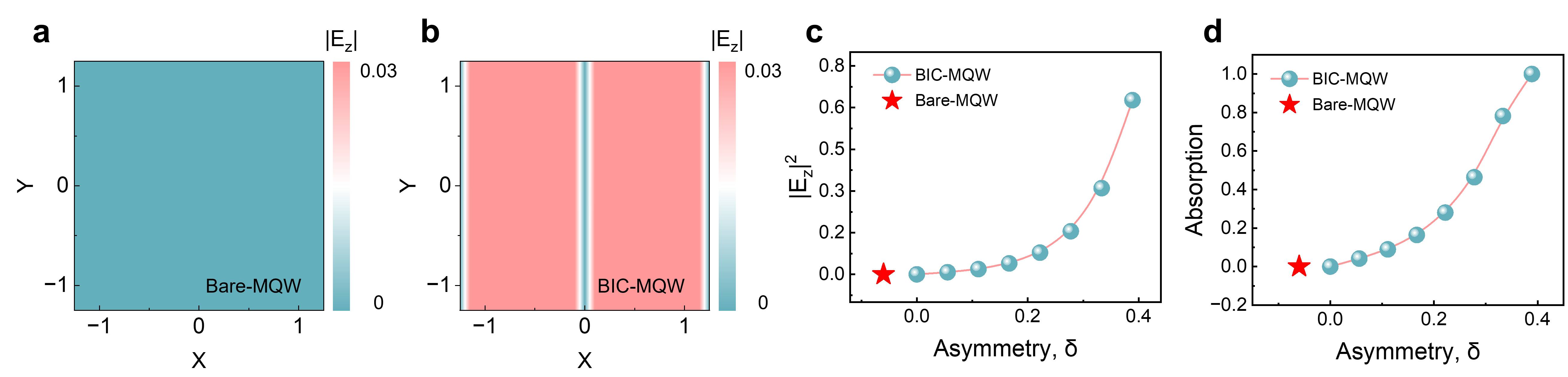
**

Fig. S9.

Simulated |***E****_z_*| (a.b), |***E****_z_*|^2^ (c) and absorption (d) for Bare-MQW and BIC-MQW devices.

**
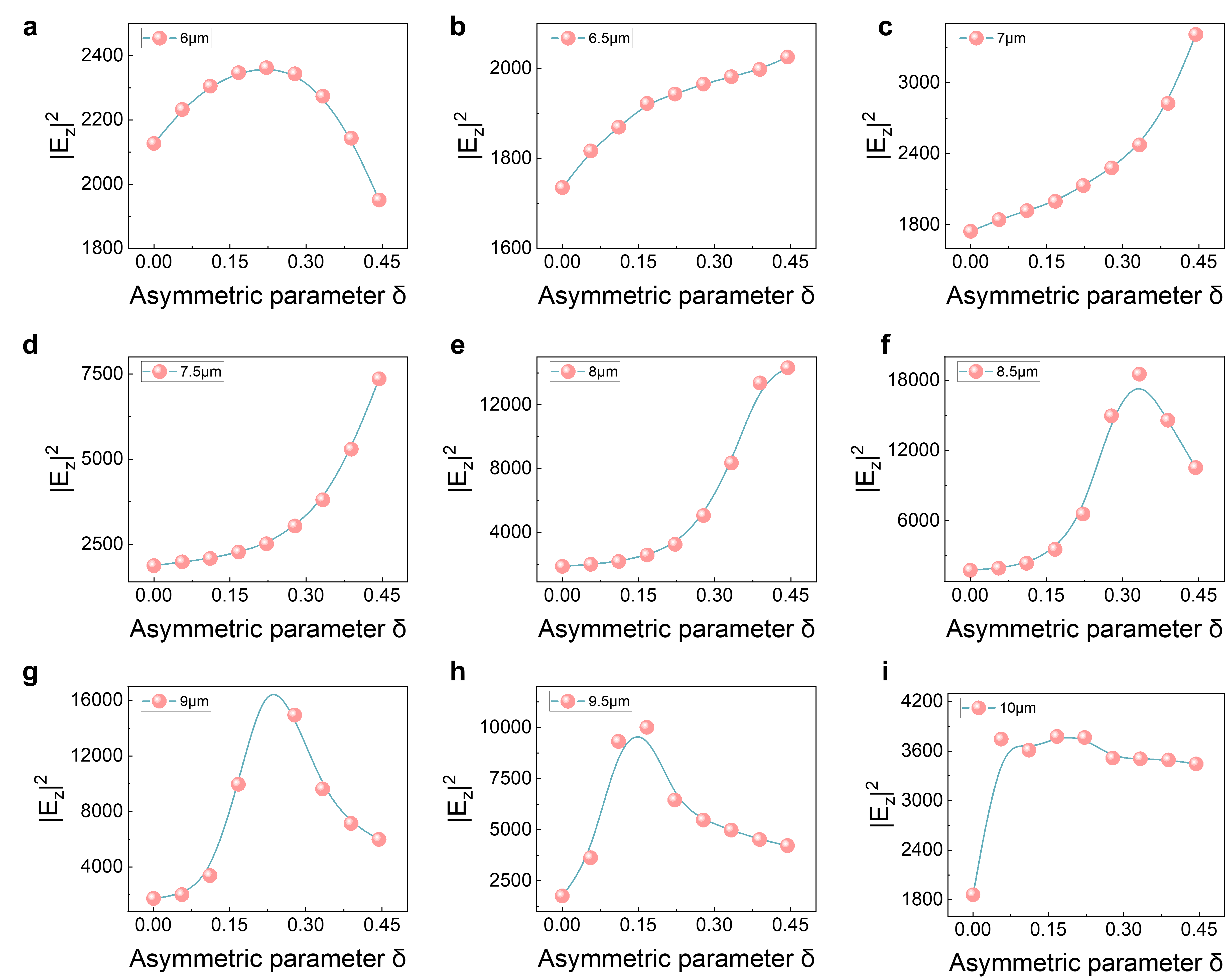
**

Fig. S10.

Simulated integral of the |***E****_z_*|^2^ at different wavelengths.

**
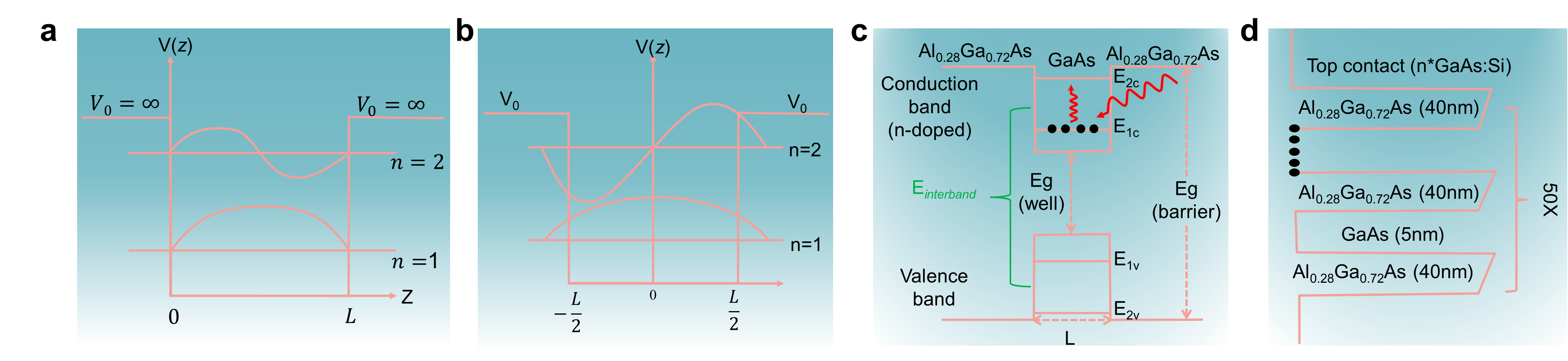
**

Fig. S11.

Theoretical analysis of MQW structure. (a) A quantum well with a width of *L* and an infinite barrier height of V_0_ = ∞, with energy levels and corresponding wave functions for *n* = 1 and *n* = 2. (b) A quantum well with a width of *L* and a finite barrier height of V_0_, with energy levels and corresponding wave functions for *n* = 1 and *n* = 2. (c) The schematic diagram of energy bands absorbed by sub bands. (d) Band diagram of the quantum well and the thicknesses of multiple the sub-layers.


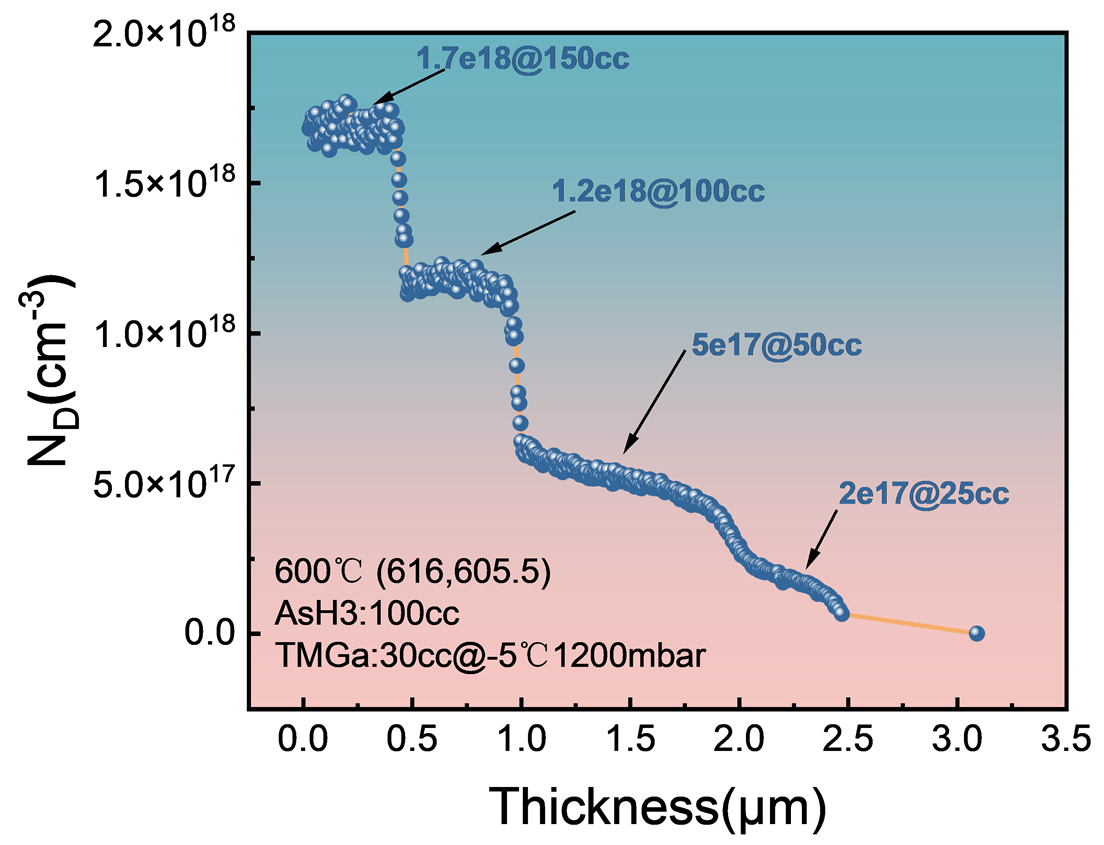


Fig. S12.

The doping concentrations were measured using ECV profiling.


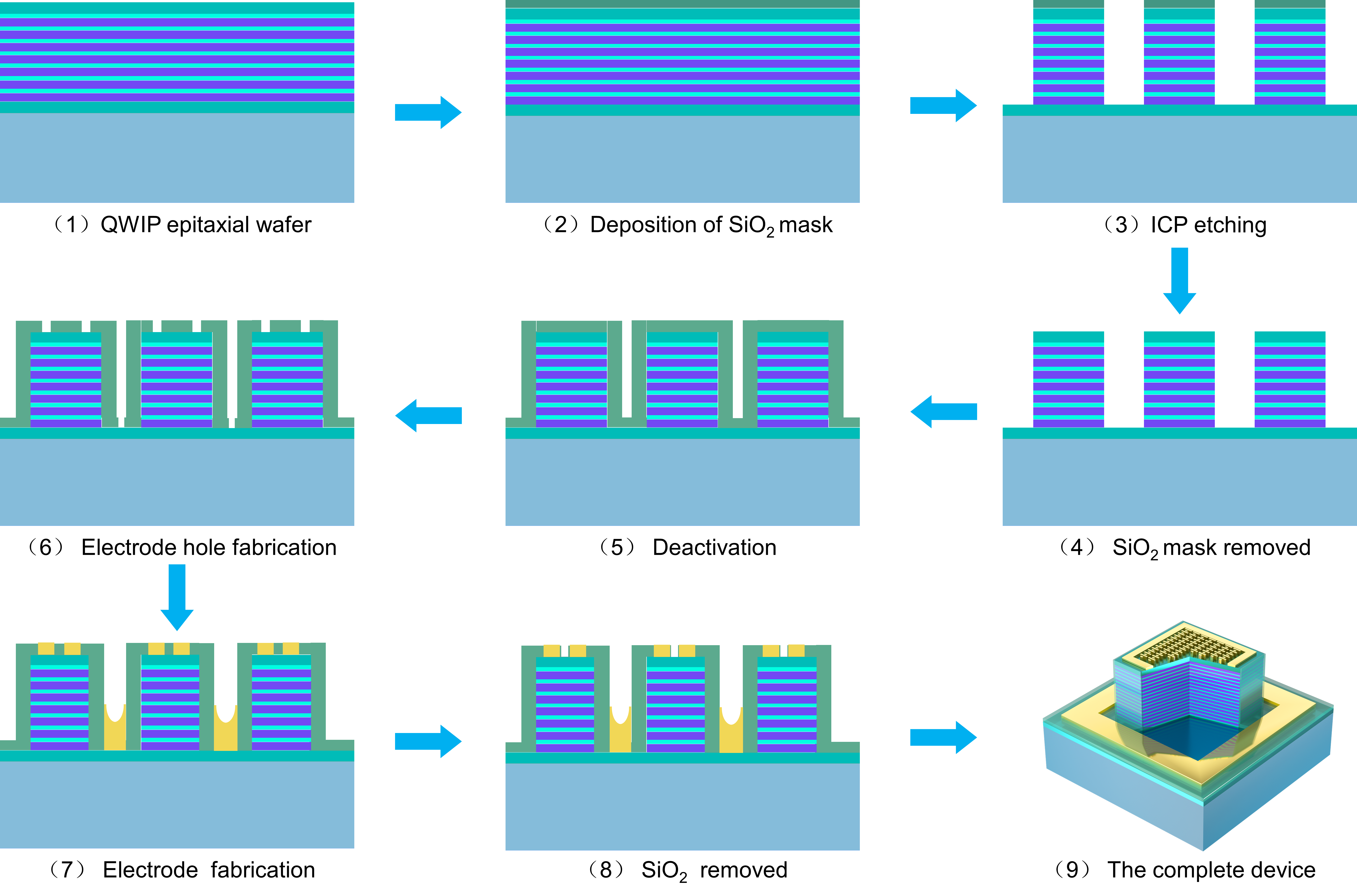


Fig. S13.

The process flow for fabricating BIC-MQW sample.


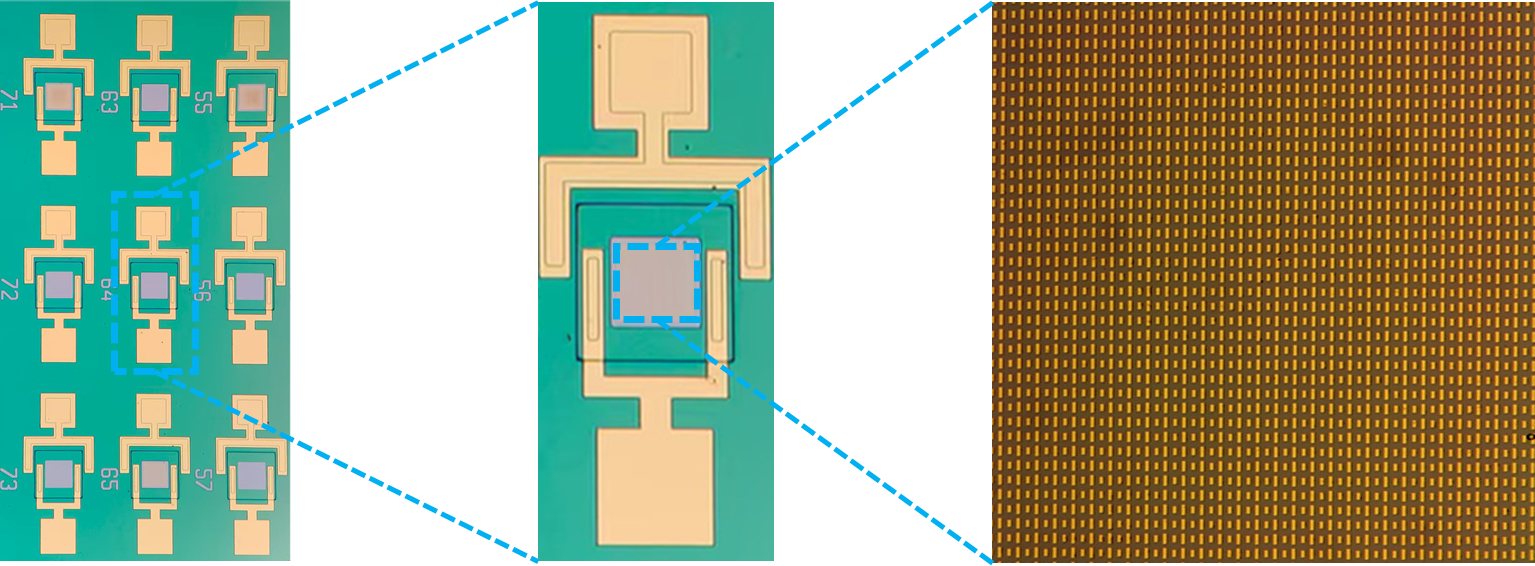


Fig. S14.

The Microscope photo of the sample.


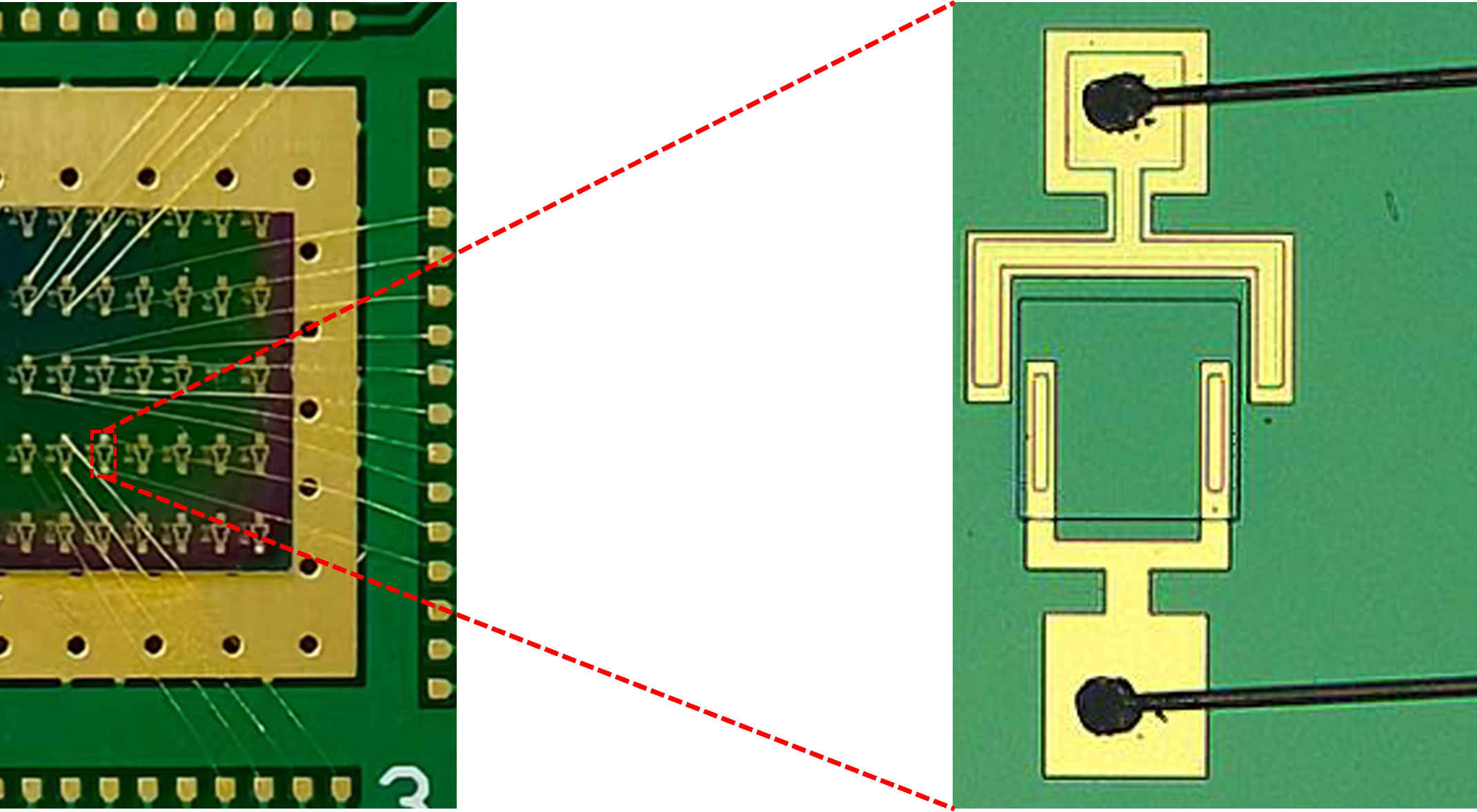


Fig. S15.

A photograph of the BIC-MQW connects with the PCB.


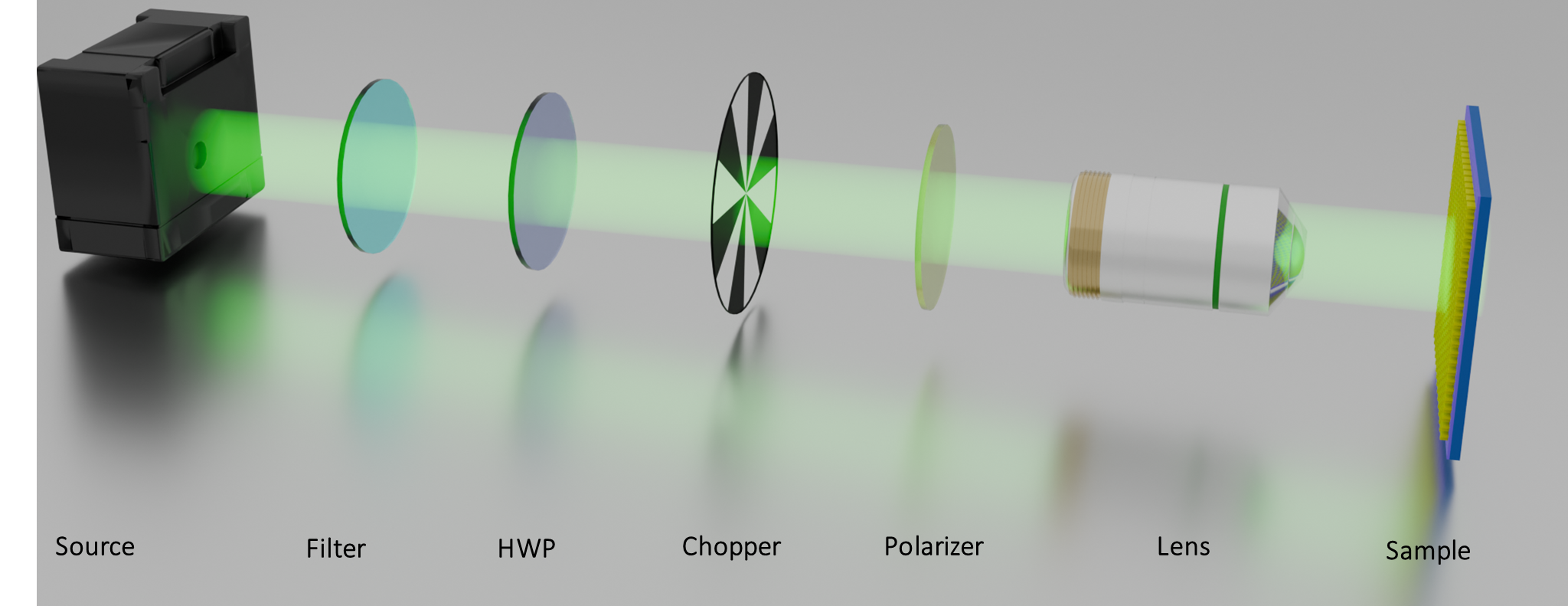


Fig. S16.

The optical pathway of experimental setup used to measure the photocurrent for BIC-MQWs sample.


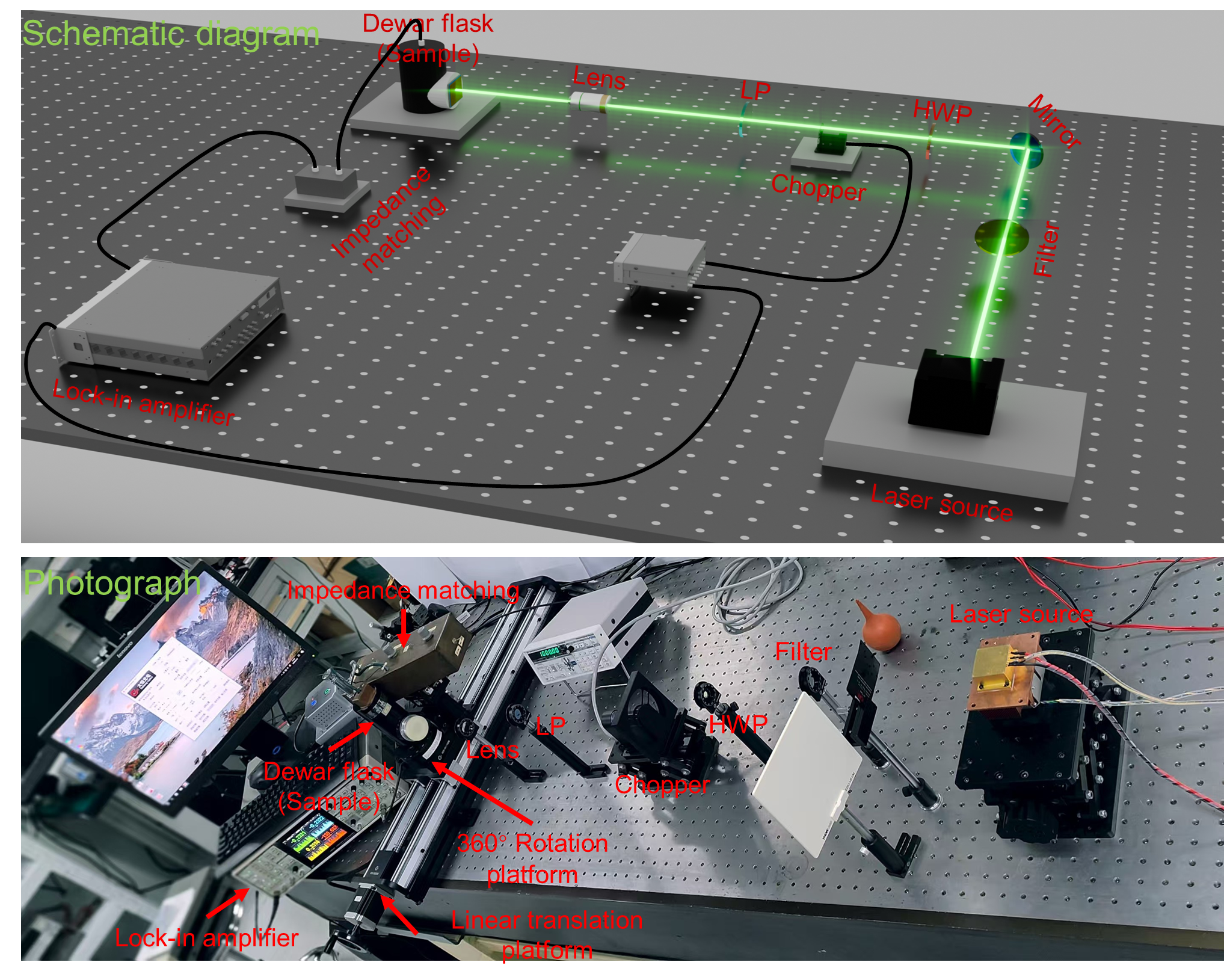


Fig. S17.

The schematic diagram and photograph of laser measurement system.


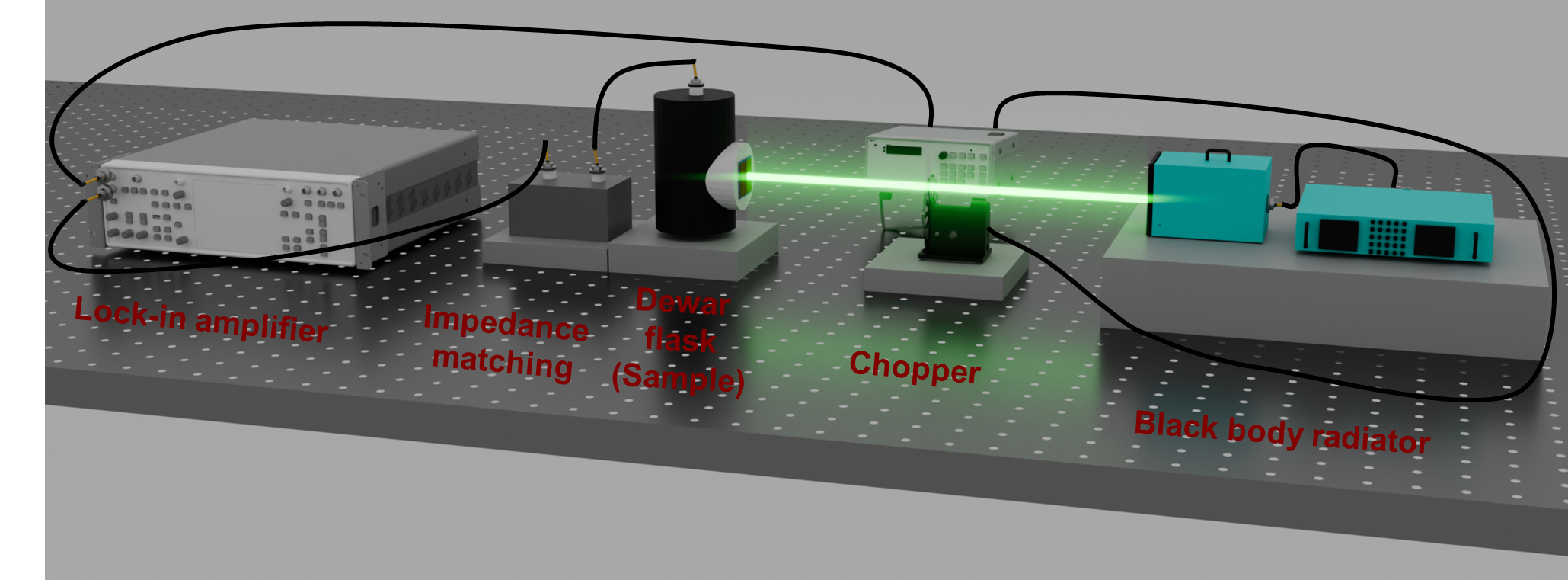


Fig. S18.

Schematic diagram of blackbody measurement system.


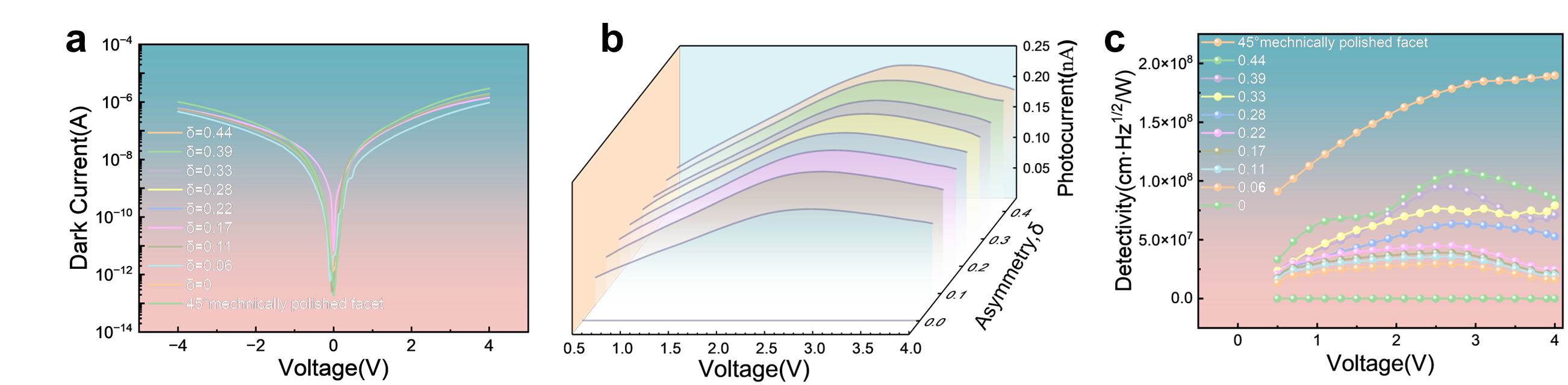


Fig. S19.

The experimental results tested by the measurement system shown in Fig. S13.


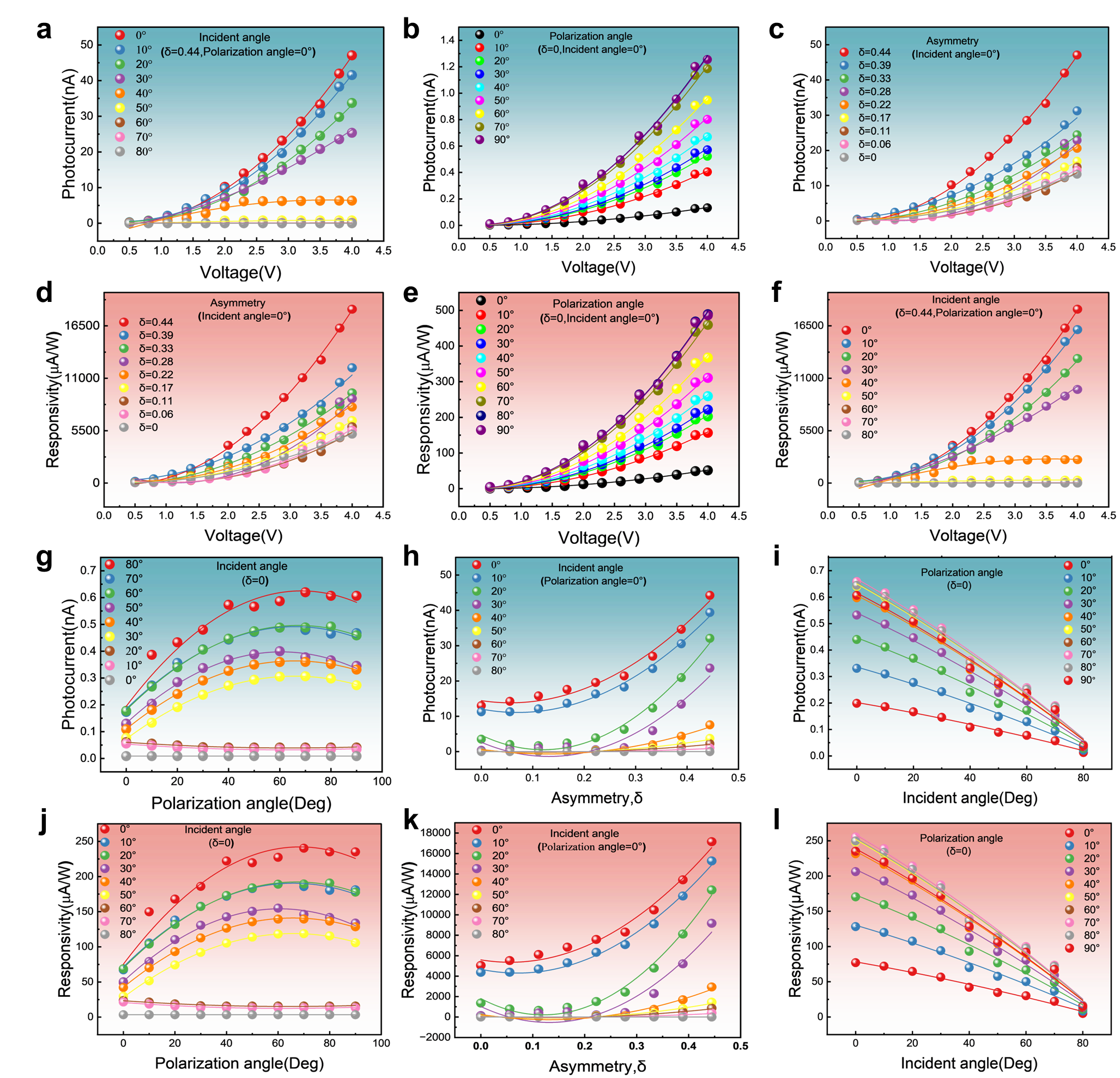


Fig. S20.

The experimental results, obtained using the measurement system in Fig. S15, characterize the dependence of the photocurrent (and thus responsivity) on three key parameters: the asymmetric parameter, the incident angle, and the applied bias voltage.


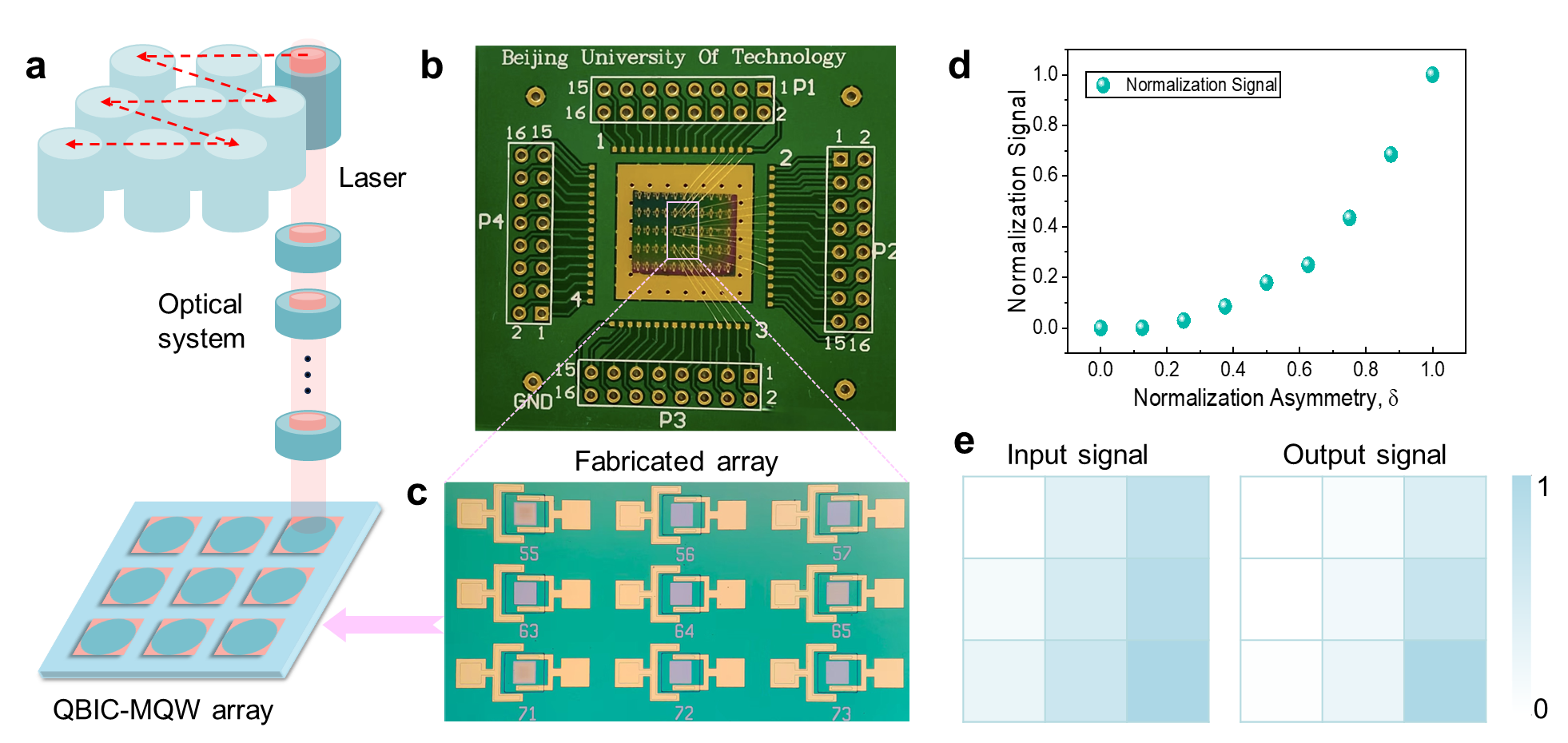


Fig. S21.

Hardware implementation of image contrast enhancement by BIC-MQWs. (a) Schematic illustration of the optical setup. (b) Macroscopic image of the bonded chip. (c) Microscope image of the BIC-MQW array, which consists of 3 × 3 pixel with nine different asymmetric parameters δ. (d) Normalized output signal (i.e., photocurrent) vs. normalized input signal (i.e., δ), where the experimental data before normalized is the same with Fig. 4e. (e) An illustration of the image contrast enhancement after the BIC-MQW preprocessing.

Fig. S22.

(a) Illustration of photoresponse-δ relationship to achieve contrast enhancement. (b) Estimation of contrast enhancement based on points A1, A2, B1 and B2.


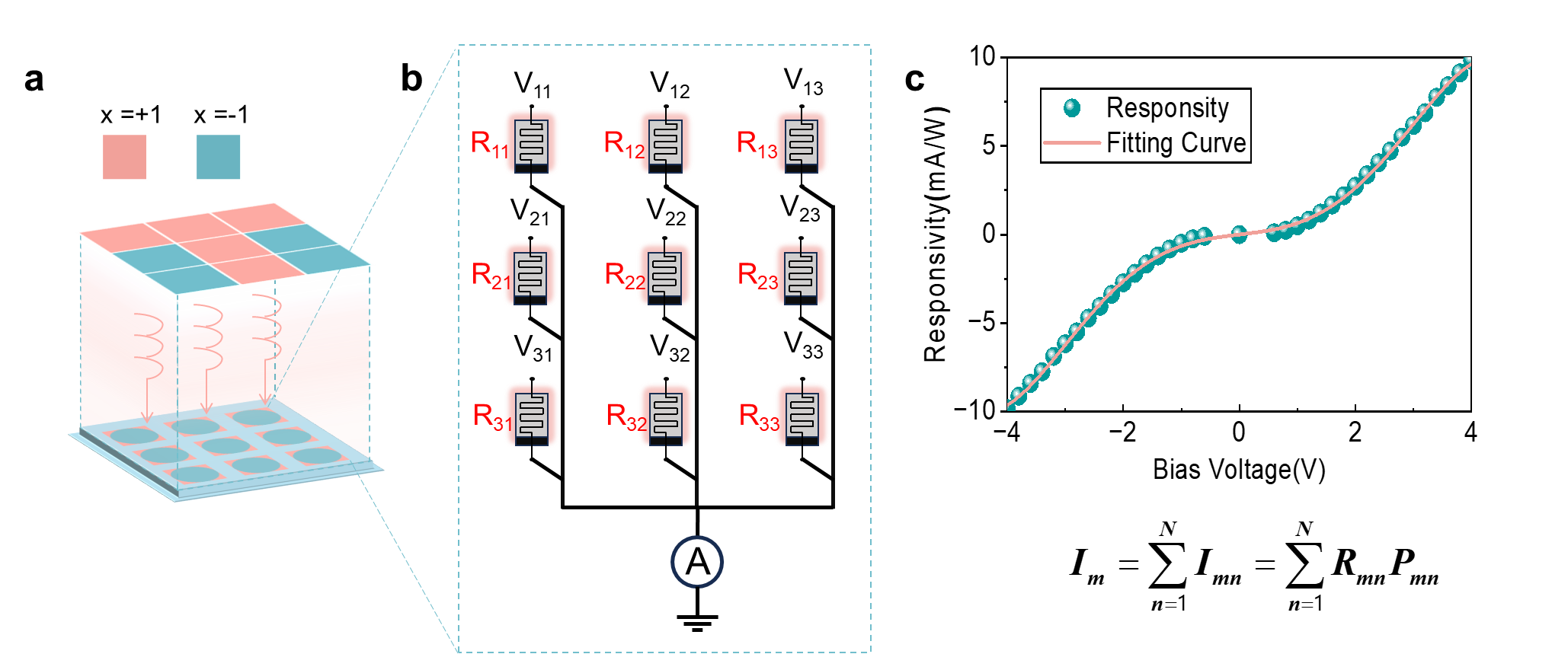


Fig. S23.

Hardware implementation of MAC operation by BIC-MQWs. (a) Schematic illustration of the MAC operation. (b) Corresponding circuit structure of a 3 × 3 BIC-MQW array. (c) Current-voltage characteristic curve of one of the BIC-MQW under optical illumination, where the experimental data is the same with Fig. 4g.


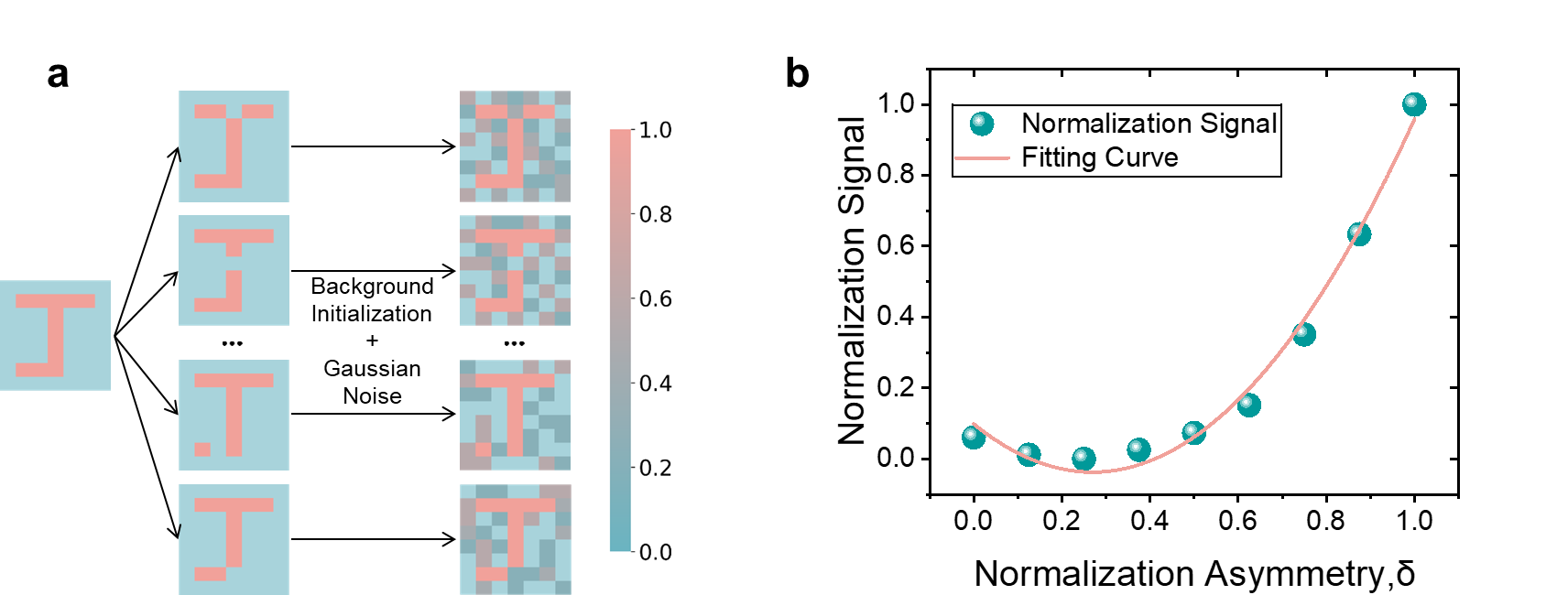


Fig. S24.

(a) The generation process of the letter ‘J’ image, in which the generated image is produced by randomly removing one main pixel from the standard ‘J’ pattern, setting the background pixel values to a uniform distribution between 0 and 0.5, and adding Gaussian noise to the image. (b) The fitted curve of the relationship between normalized asymmetry and normalized signal, where the experimental data before normalized is the same with Fig. 4e.


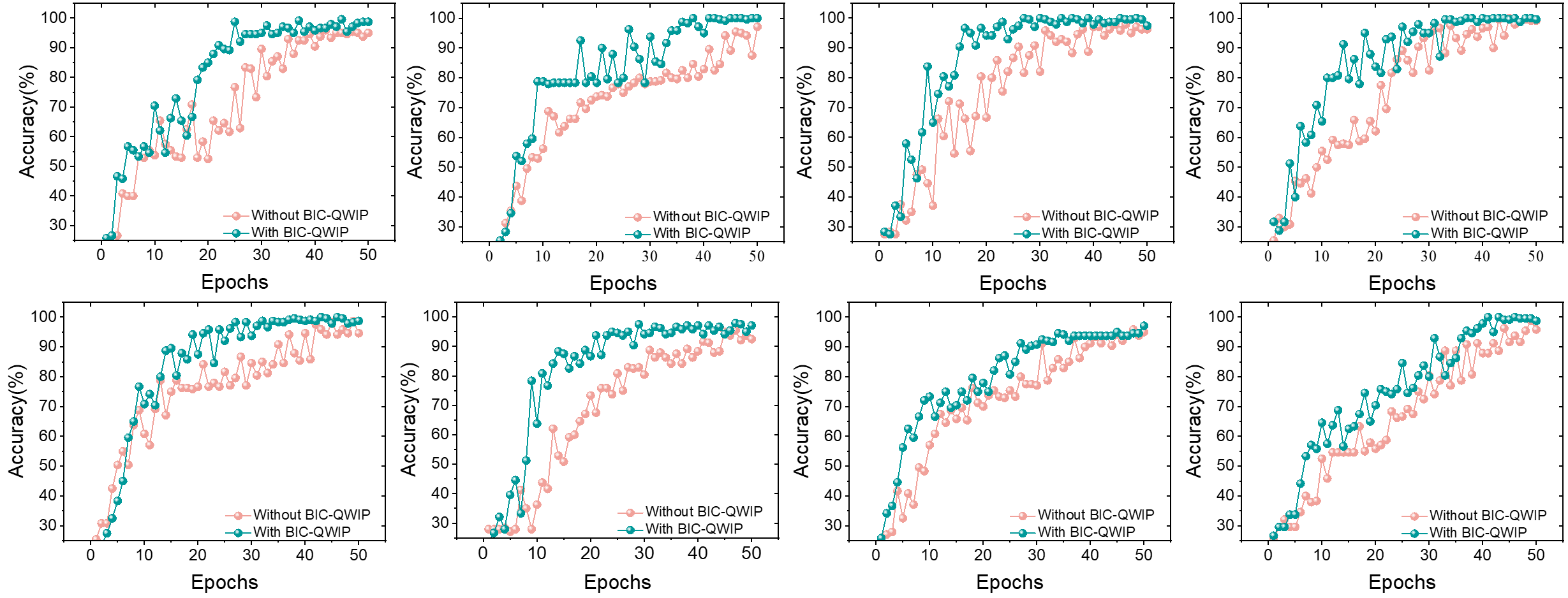


Fig. S25.

Comparison of different random seeds generate different image data. The training process with enhanced contrast converges faster in terms of accuracy compared to the original image set.


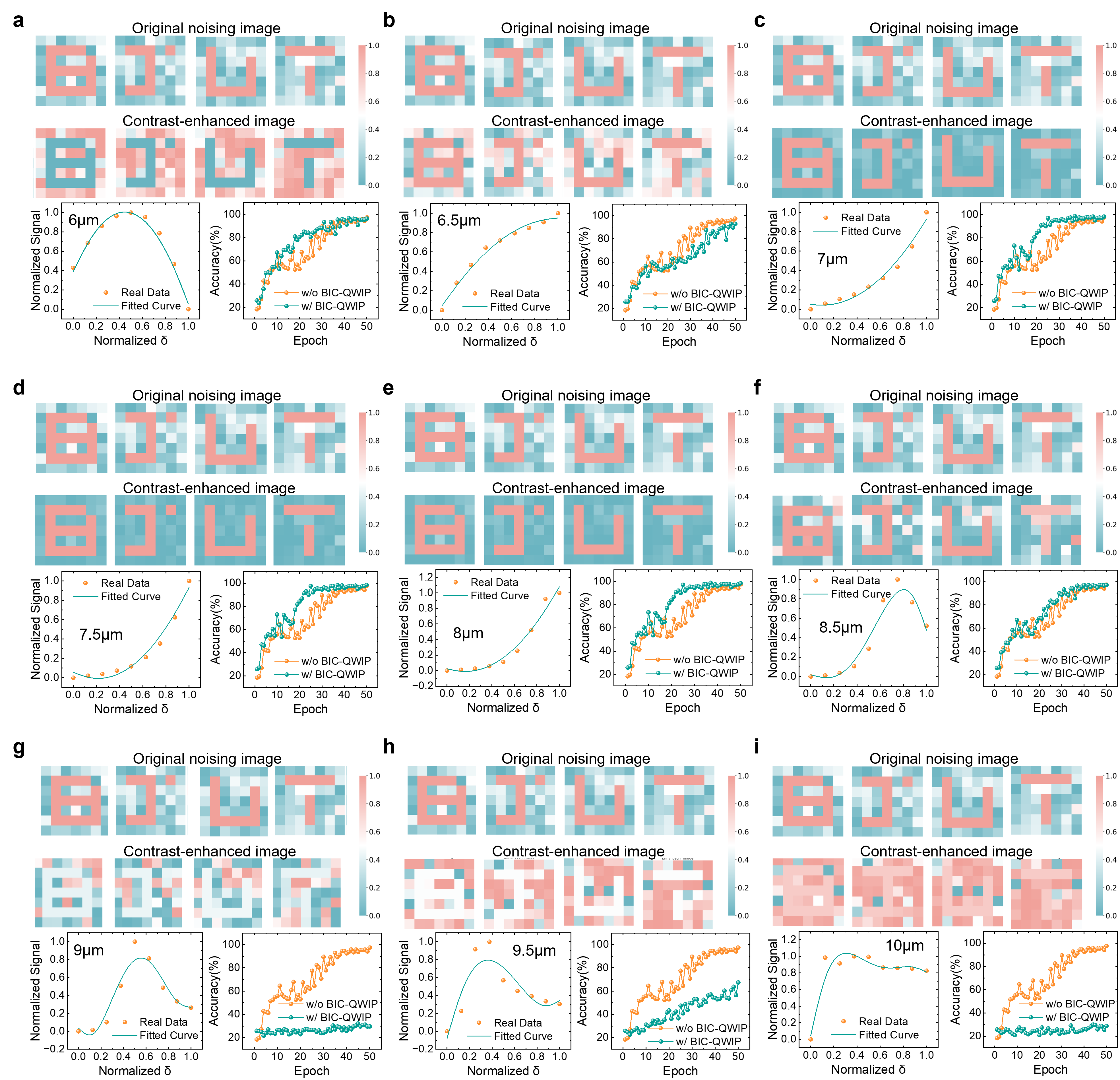


Fig. S26.

Simulated wavelength-dependent recognition performance based on the QBIC-mediated |***E****_z_*|^2^ response in Fig. R14 from 6μm to10μm, including comparison of images before and after BIC-MQW-based preprocessing and corresponding recognition accuracy during training epochs with and without BIC-MQW-based preprocessing.


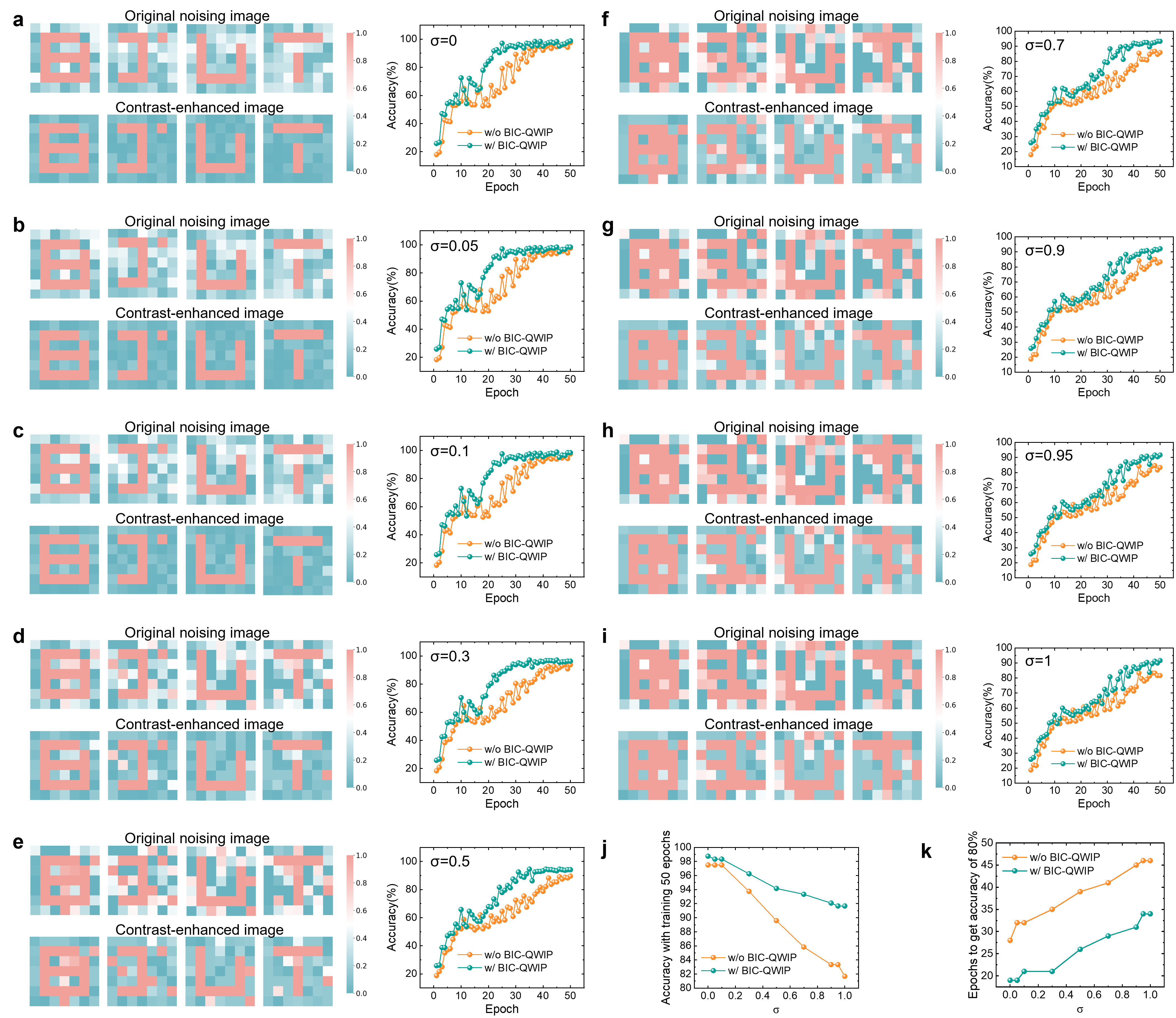


Fig. S27.

Simulations of SNR-dependent image recognition in a neuromorphic visual system with BIC-MQW, where different noise of background pixel value following a uniform distribution U(a_U_, b_U_) is considered by setting a_U_=0 and b_U_ ranges from 0 to 1. Comparison of image before and after BIC-MQW-based preprocessing and corresponding recognition accuracy during training epochs with respect to (a) b_U_ = 0, (b) b_U_ = 0.1, (c) b_U_ = 0.2, (d) b_U_ = 0.3, (e) b_U_ = 0.4, (f) b_U_ = 0.5, (g) b_U_ = 0.6, (h) b_U_ = 0.7, (i) b_U_ = 0.8, (j) b_U_ = 0.9, and (k) b_U_ = 1. (l) Recognition accuracy as a function of b_U_ with and without BIC-MQW-based preprocessing, when training epochs reach 50. (m) Number of training epoch as a function of b_U_ with and without BIC-MQW-based preprocessing, when recognition accuracy reaches 80%.


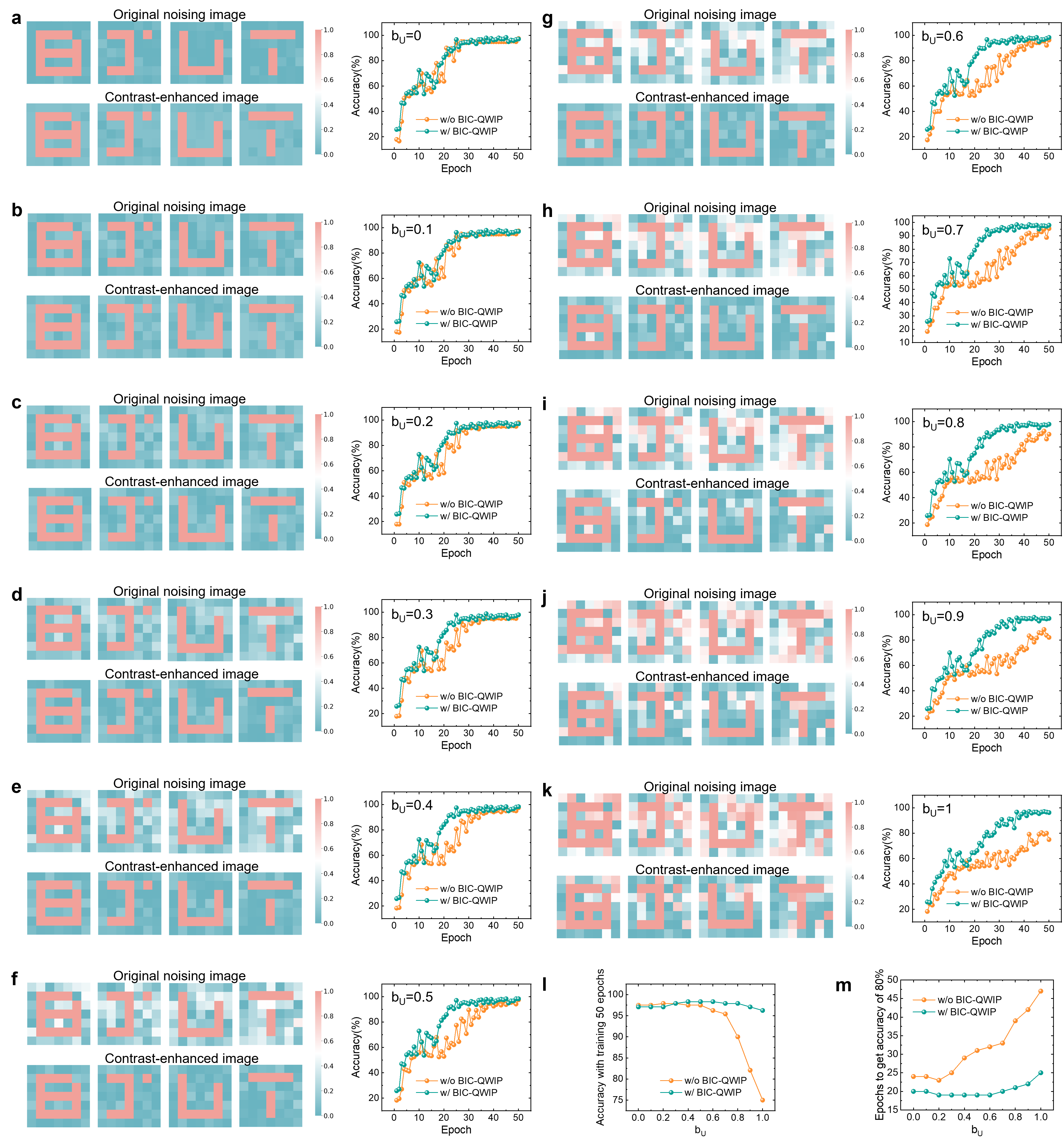


Fig. S28.

Simulations of SNR-dependent image recognition in a neuromorphic visual system with BIC-MQW, where different noise of both main body pixel and background pixel values following a Gaussian distribution Gaussian noise N(μ, σ^2^) is considered by setting μ=0 and σ ranges from 0 to 1. Comparison of image before and after BIC-MQW-based preprocessing and corresponding recognition accuracy during training epochs with respect to (a) σ = 0, (b) σ = 0.05, (c) σ = 0.1, (d) σ = 0.3, (e) σ = 0.5, (f) σ = 0.7, (g) σ = 0.9, (h) σ = 0.95, and (i) σ = 1. (j) Recognition accuracy as a function of σ with and without BIC-MQW-based preprocessing, when training epochs reach 50. (k) Number of training epoch as a function of σ with and without BIC-MQW-based preprocessing, when recognition accuracy reaches 80%.


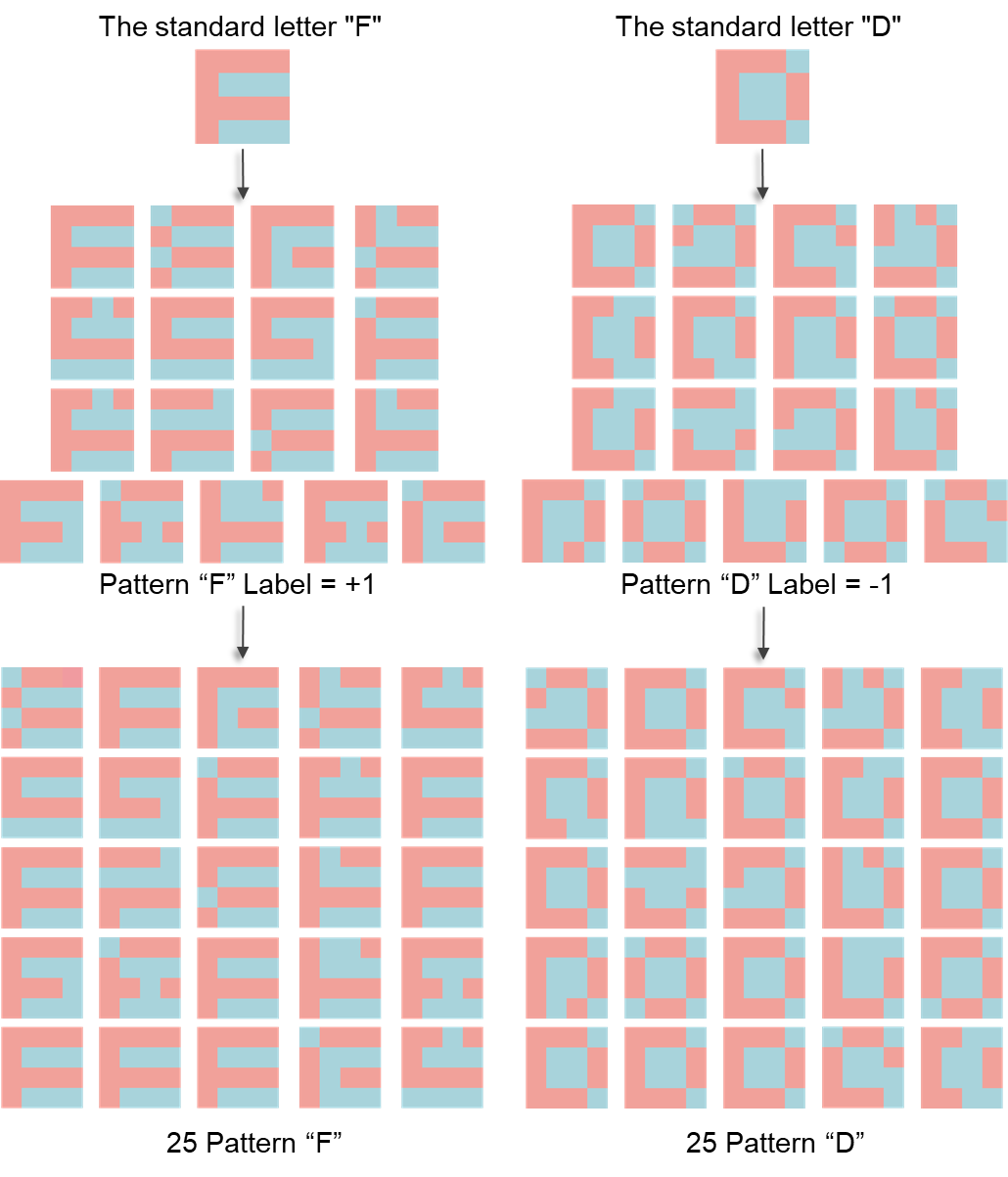


Fig. S29.

A dataset comprising 17 distinct patterns for each of the two letters is constructed. These 17 different patterns (which include the standard patterns) are derived from the pixel arrays of the standard letters ‘F’ and ‘D’, with their respective target values set to +1 and -1. The generated dataset consists of 50 4×4 pixel arrays, with the first 25 representing the ‘F’ pattern (as shown in the lower left figure) and the last 25 representing the ‘D’ pattern (as shown in the lower right figure).


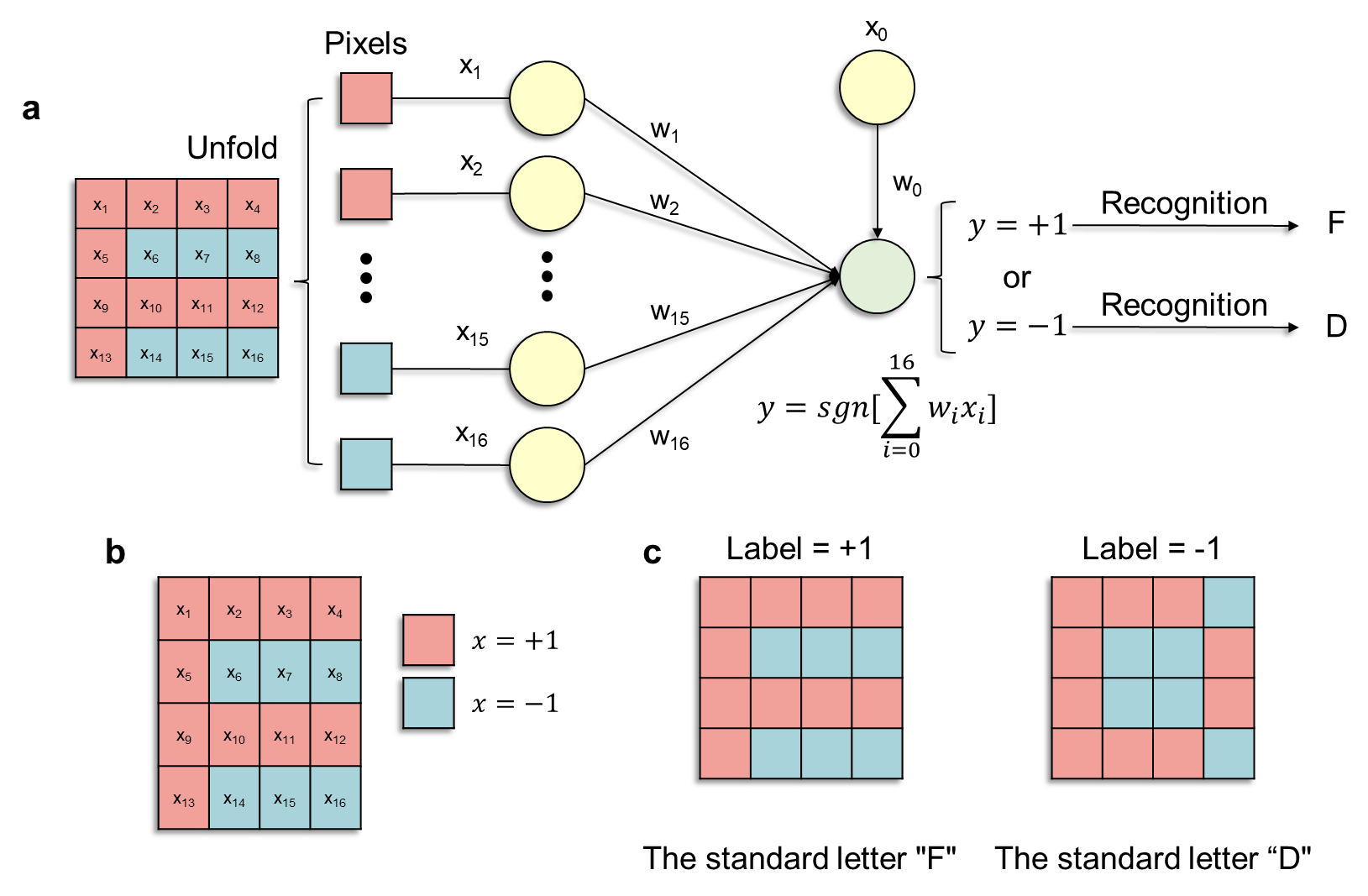


Fig. S30.

Letter recognition from input pixel arrays. (a) A binarized 4×4 pixel array is fed into a single-layer perceptron, and the classification of the input data is determined by the logical output value of the output neuron, thereby identifying the type of letter. (b) The binarized logical values of the input 4×4 pixel array, where dark pixels correspond to +1 and light pixels correspond to -1. (c) The pixel arrays for the standard patterns of the letters ‘F’ and ‘D’ along with their respective target values.

Fig. S31.

Implementation of input image with N×N pixels mapped onto M×M sensor array for MAC operation: (a) M>N, (b) M=N, and (c) M>N.


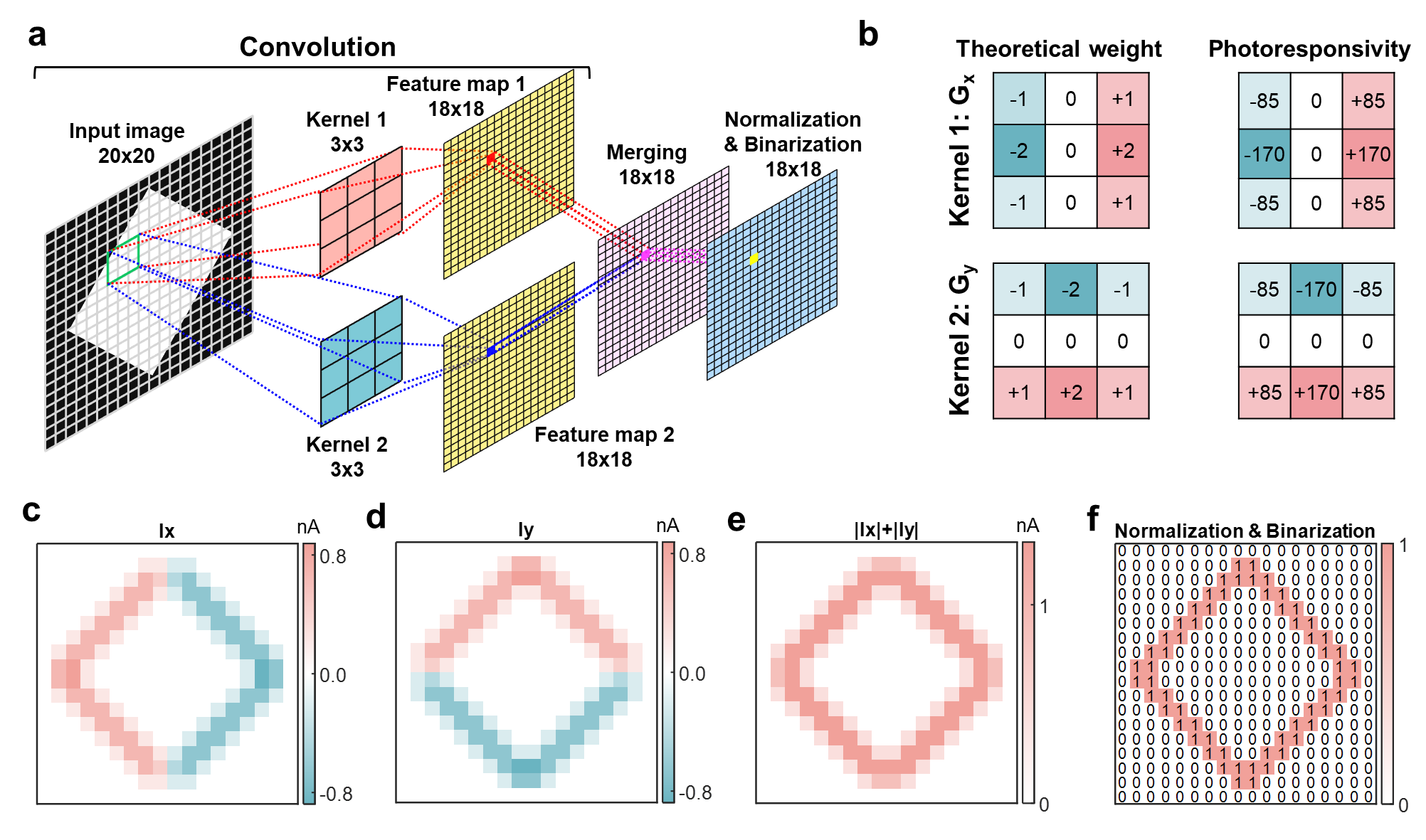


Fig. S32.

Image processing of Edge detection. (a) Schematic illustration of edge detecting process. (b) From left to right: theoretical dimensionless weights, photoresponsivity weight in unit [μA W^-1^] generated by the QBIC-MQW devices, where the mapping relationship of photoresponsivity to incident angle is taken from Fig. 4f. (c) and (d): edge gradient components for *x* direction (I*_x_*) and *y* direction (I*_y_*), respectively. (e) Output result after merging I*_x_* and I*_y_*. (f) Final output after normalization and binarization.

**
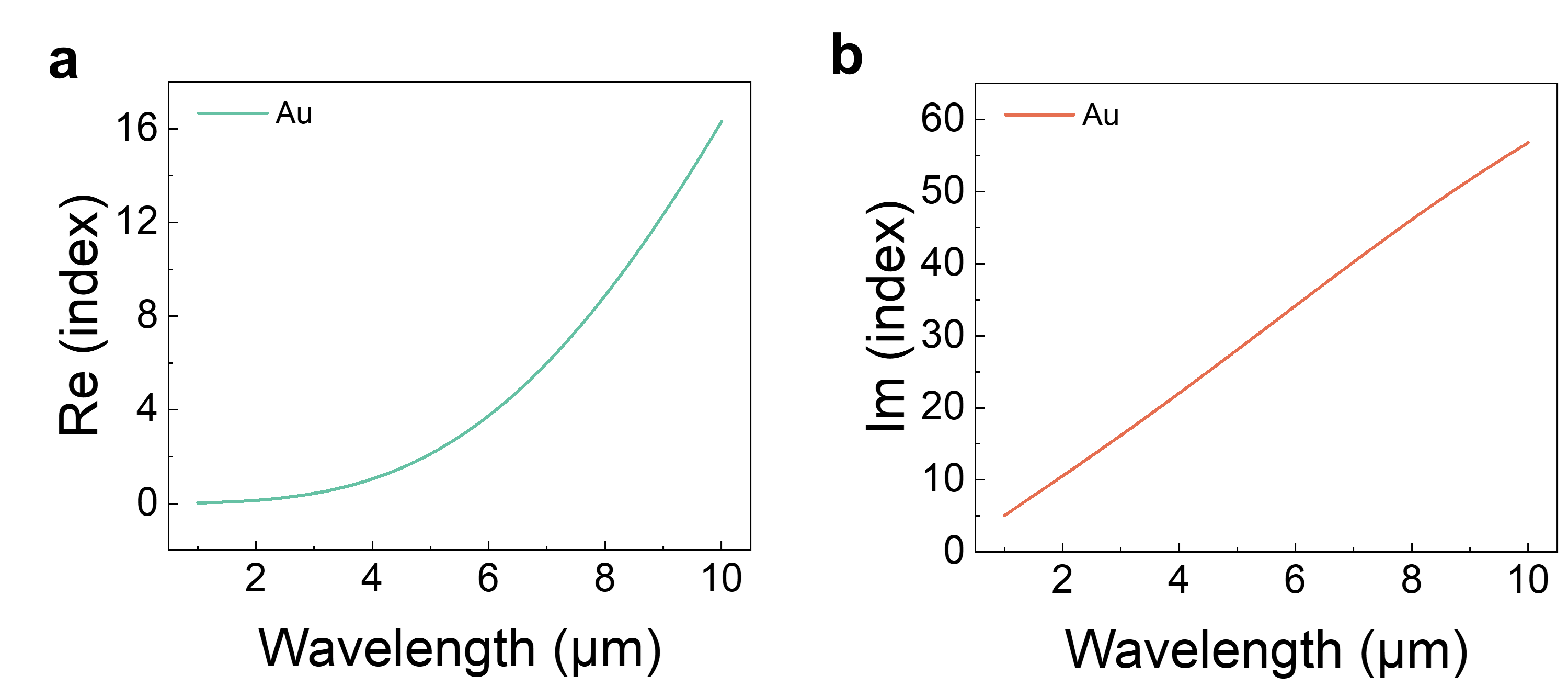
**

Fig. S33.

Characteristics of metal materials at different wavelengths.

**
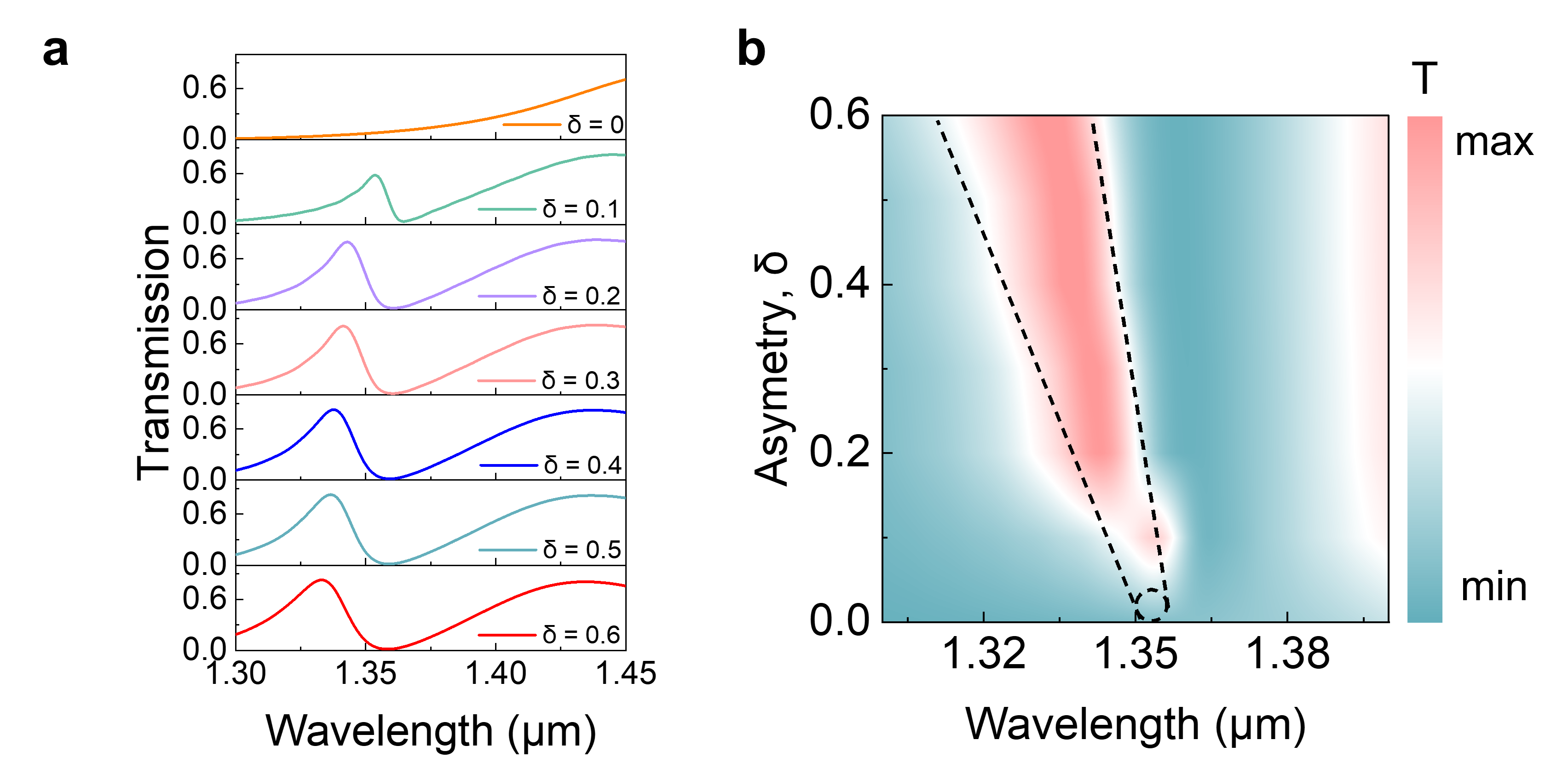
**

Fig. S34.

Transmission at short wavelengths (~1.3 μm).

Table S1.

The contrast of the BIC-MQW device with other prior MS-enabled (or BIC-enabled) optoelectronics.

Table S2.

The corresponding Pearson correlation coefficients for both fitting methods.

Table S3.

Epoch number for the image recognition tasks with and without BIC-MQW-based preprocessing. The improvement rate in epoch number with the inclusion of BIC-MQW are also listed in the table.


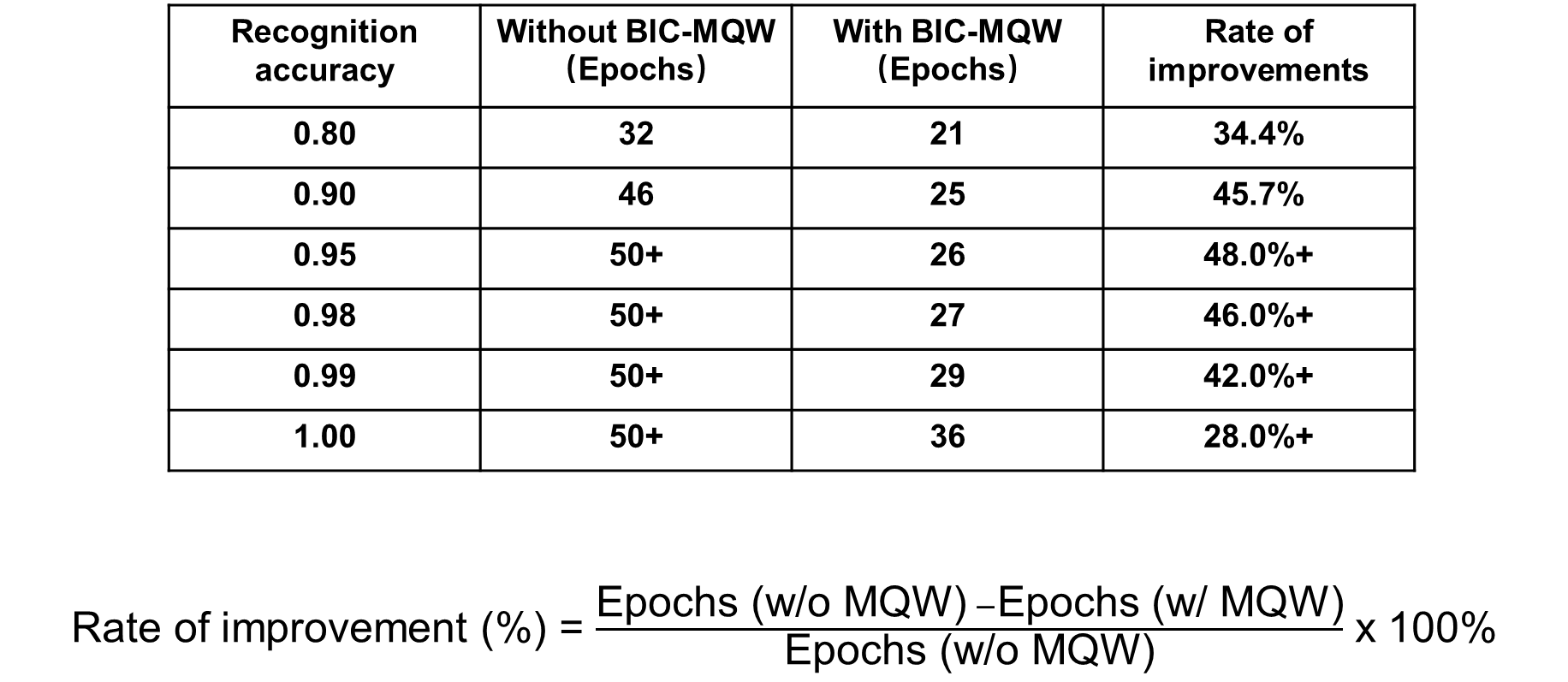

Supplement: Supplementary file 1 — Supplementary Information for Quasi-bound States in the Continuum Driven Photoresponse in Multiple Quantum Wells for Machine Vision [file 41377_2026_2404_MOESM1_ESM.docx]
